# An inhibitory GLP-1 circuit in the lateral septum modulates reward processing and alcohol intake in rodents

**DOI:** 10.1016/j.ebiom.2025.105684

**Published:** 2025-04-17

**Authors:** Christian E. Edvardsson, Davide Cadeddu, Mia Ericson, Louise Adermark, Elisabet Jerlhag

**Affiliations:** a Department of Pharmacology, Institute of Neuroscience and Physiology, The Sahlgrenska Academy, University of Gothenburg, Gothenburg, Sweden; b Addiction Biology Unit, Department of Psychiatry and Neurochemistry, Institute of Neuroscience and Physiology, The Sahlgrenska Academy, University of Gothenburg, Gothenburg, Sweden

**Keywords:** Lateral septum, GLP-1, Alcohol, Reward, Dopamine, GABA

## Abstract

**Background:**

Alcohol use disorder (AUD) is a complex psychiatric condition with limited effective treatment options. Glucagon-like peptide-1 receptor (GLP-1R) agonists have emerged as potential AUD treatment, as they have been shown to modulate reward-related behaviours, including those linked to alcohol consumption. However, the underlying mechanisms and neurocircuitry remain unclear. This study investigated the role of GLP-1R in the lateral septum (LS), a brain region highly expressing GLP-1R and implicated in reward-related behaviours, including alcohol-induced reward and consumption.

**Methods:**

Behavioural, neurochemical, molecular, and electrophysiological methods were used to investigate the effect of LS GLP-1R signalling in alcohol-mediated responses in rodents.

**Findings:**

LS GLP-1R activation attenuated alcohol’s rewarding effects, reducing locomotor stimulation, place preference, and accumbal dopamine release. Intra-LS infusion of the GLP-1R agonist exendin-4 (Ex4) reduced alcohol intake dose-dependently without affecting food or water consumption, while GLP-1R inhibition increased alcohol intake. Furthermore, LS GLP-1R expression correlated with alcohol intake in male but not female rats, suggesting sex-specific effects of long-term alcohol exposure. *Ex vivo* electrophysiology indicated that GLP-1R activation depressed LS neurotransmission via a gamma-aminobutyric acid (GABA)_A_ receptor-dependent mechanism.

**Interpretation:**

This study provides new insights into how GLP-1R agonists may reduce alcohol intake. Overall, the findings underscore the potentially inhibitory neuromodulatory role of LS GLP-1R in regulating alcohol consumption through the modulation of dopaminergic reward processes tentatively involving GABA transmission.

**Funding:**

10.13039/501100004359Swedish Research Council (2023-2600), 10.13039/501100005754Sahlgrenska University HospitalLUA/ALF (grant no. 723941), 10.13039/501100014552Adlerbertska Research Foundation and Professor Bror Gadelius Foundation.

**Research in context**

**Evidence before this study**

Glucagon-like peptide-1 receptor (GLP-1R) agonists have emerged as potential treatments for alcohol use disorder (AUD). Previous studies have shown that GLP-1R agonists can reduce alcohol consumption in rodents and nonhuman primates, as well as modulate alcohol-related behaviours. Specifically, GLP-1R agonists like exendin-4, liraglutide, and semaglutide have been found to decrease alcohol intake, relapse-like drinking, and alcohol-induced reward processes in animal models. Clinical support is provided by randomised clinical trials, register-based studies and case–control studies suggesting that GLP-1R agonists also may reduce alcohol consumption in humans. Research from various groups has indicated that the lateral septum (LS), a brain region with high GLP-1R expression, is involved in modulating reward processes related to food, drugs, and alcohol. However, the precise neurobiological mechanisms and sex-specific differences in GLP-1R-mediated effects on alcohol-related responses in the LS remain to be elucidated.

**Added value of this study**

This study builds upon the existing knowledge by providing mechanistic insights into the role of GLP-1R signalling in the LS. The findings demonstrate that LS GLP-1R activation attenuates alcohol's rewarding effects, reduces alcohol intake, and suppresses accumbal dopamine release after alcohol in rodents. The study also reveals sex-specific effects of long-term alcohol exposure on LS GLP-1R expression and identifies a potential GABA_A_-receptor-dependent mechanism through which GLP-1R activation depresses LS neurotransmission. These results expand our understanding of the neurocircuitry involved in GLP-1R-mediated modulation of alcohol consumption and reward processes, highlighting the LS as a region of interest in this system.

**Implications of all the available evidence**

The findings in this study provide a deeper mechanistic insight into the effects of GLP-1R activation on alcohol-related responses in the LS, complementing existing literature on the subject. As we show that GLP-1R in the LS regulates alcohol-related responses, this adds to the growing body of evidence supporting the clinical use of GLP1-R agonists as AUD treatment. Future studies should further explore the specific GABA_A_-receptor-dependent mechanisms in LS GLP-1R signalling and investigate how GLP-1R agonists, such as semaglutide, modulate LS activity in patients with AUD. Additionally, the observed sex-specific effects in LS GLP-1R expression underscore the need for further investigation into potential differences in treatment responses between sexes, to optimise treatment outcomes.

## Introduction

Alcohol use disorder (AUD) poses a significant global public health challenge owing to its complex psychiatric nature.^1^^,^^2^ Currently, approved pharmacological treatments for AUD are associated with varying efficacy, emphasising the urgent need for new therapeutic strategies.^3^^,^^4^ However, developing effective medications for individuals with AUD requires a deeper understanding of the neurobiological mechanisms underlying excessive alcohol consumption and the progression to dependence. Several neurotransmitters are involved in these processes, with the dopaminergic reward system as a key component.^5^^,^^6^ The release of dopamine in the nucleus accumbens (NAc) promotes the immediate reinforcing effects of alcohol, leading to a feeling of euphoria and a learnt association between alcohol and the behaviour that caused it.^5^^,^^6^ This reward-related reinforcement contributes to increased alcohol consumption and elevates the risk of developing AUD later in life.^7^, ^8^, ^9^, ^10^, ^11^, ^12^ Importantly, emerging evidence has emphasised the role of gut-brain peptides in mediating these processes, making them promising candidates for treating AUD.^13^, ^14^, ^15^ One such peptide is glucagon-like peptide-1 (GLP-1) and its receptor (GLP-1R).^15^, ^16^, ^17^

GLP-1R is widely expressed throughout the brain,^18^, ^19^, ^20^, ^21^ and several studies show that GLP-1R in reward processing areas, such as the ventral tegmental area (VTA), laterodorsal tegmental area (LDTg), and NAc influences both consummatory and reward-related behaviours.^22^, ^23^, ^24^, ^25^, ^26^, ^27^, ^28^, ^29^, ^30^, ^31^ In fact, agonists that target these receptors, such as exendin-4 (Ex4), liraglutide, and semaglutide, reduce alcohol intake and suppress alcohol-induced activation of the mesolimbic dopamine system in rodents and nonhuman primates.^31^, ^32^, ^33^, ^34^, ^35^, ^36^, ^37^, ^38^ Clinical evidence for effects in humans is so far provided by randomised clinical trials, register studies and case–control studies that indicate the GLP-1R agonists reduce alcohol intake,^39^, ^40^, ^41^, ^42^, ^43^, ^44^, ^45^, ^46^, ^47^ but additional randomised clinical trials are currently underway to explore if GLP-1R agonists potentially could be used as a treatment option for AUD. However, the precise mechanisms and neurocircuitry through which GLP-1R influences these processes remain poorly understood.

One brain region of particular interest with regards to reward processing is the lateral septum (LS), which interestingly displays high expression of GLP-1R.^18^, ^19^, ^20^, ^21^ The LS is recognised as a key relay and integration hub within the medial forebrain^48^, ^49^, ^50^, ^51^ with extensive connections to multiple substrates of the reward system. Owing to this, the LS is thought to play a central role in the regulation of affective, motivational, and reward-related behaviours.^48^, ^49^, ^50^, ^51^, ^52^ Importantly, the LS modulates appetitive and consummatory behaviours associated with food and drugs of abuse, including alcohol.^30^^,^^53^, ^54^, ^55^, ^56^, ^57^, ^58^, ^59^, ^60^, ^61^ Furthermore, the LS has been implicated in the control of alcohol-induced dopamine release in the NAc,^58^ possibly through gamma-aminobutyric acid (GABA)ergic projections to the VTA, or NAc.^48^^,^^54^^,^^58^^,^^62^^,^^63^

Previous studies have demonstrated that GLP-1R in the LS is expressed on GABAergic neurons,^21^^,^^64^ and is involved in regulating motivational behaviours.^30^^,^^55^^,^^56^^,^^64^ A recent study showed that activation of LS GLP-1R reduces alcohol self-administration in mice,^30^ and GLP-1R agonists such as Ex4 and semaglutide, which reach the LS,^30^^,^^65^^,^^66^ decrease septal cue reactivity to alcohol in patients with AUD.^46^ This highlights the potential role of GLP-1R in the LS in alcohol-induced reward and alcohol consumption. However, the neurobiological mechanisms involved, particularly those related to GABAergic signalling, remain to be elucidated.

In the present study, we sought to address these knowledge gaps by initially exploring if LS GLP-1R is involved in mediating the systemic effects of Ex4. Secondly, we wanted to explore the role of LS GLP-1R in reward processes, focusing on alcohol-induced reward responses, and alcohol consumption. Lastly, we investigated whether a GABAergic mechanism could be involved in mediating these effects. By clarifying the aforementioned processes, this study aims to contribute to a more comprehensive understanding of LS GLP-1R’s role in alcohol consumption and reward processing in rodents, as well as to explore factors, such as sex, that potentially could affect therapeutic outcomes of GLP-1R agonists.

## Methods

### General research outline

As summarised in Table 1, a combination of experiments was used to decipher the role of LS GLP-1Rs in alcohol consumption and define the mechanisms involved in these effects.

**Table 1:** Overview of the experimental experiments.

This table summarizes the range of behavioural, neurochemical, molecular, and electrophysiological methodologies employed to elucidate the role of LS GLP-1R in modulating alcohol consumption and explore possible involved mechanisms of these effects. The experiments were conducted on both male (M) and female (F) subjects.

Lateral septum (LS), lateral ventricles (ICV), caudate putamen (CPu), nucleus accumbens (NAc), ventral tegmental area (VTA), laterodorsal tegmental area (LDTg), locomotor activity (LMA), conditioned place preference (CPP), intermittent access alcohol drinking studies (IA2BC), field potential recordings (EP), population spike (PS), paired-pulse ratio (PPR), gene expression (qPCR), glucagon-like peptide-1 receptor (GLP-1R), vehicle (Veh), alcohol (Alc), exendin-4 (Ex4), exendin-9 (Ex9), bicuculline (Bic), artificial cerebrospinal fluid (aCSF), noradrenaline (NA), serotonin (5-HT), 3,4-dihydroxyphenylacetic acid (DOPAC), 3-methoxy-4-hydroxyphenethylamine (3-MT), homovanillic acid (HVA), normetanephrine (NM), 5-hydroxyindoleacetic acid (5-HIAA), L-β-3,4-dihydroxyphenylalanine (L-DOPA), supplementary figure (SF). Animations were created with BioRender.

### Animals

Adult male naval medical research institute (NMRI) mice (weighing 25–30 g at arrival; Charles River, Sulzfeld, Germany) were used for locomotor activity and conditioned place preference (CPP) tests. Adult male and female Rcc/Han Wistar rats (weighing 180–250 g on arrival; Envigo, Horst, Netherlands) were used for locomotor activity, microdialysis, alcohol intake, gene expression and *ex vivo* electrophysiology experiments. The mice and rat strains used in the present study have previously been shown to exhibit robust behavioural responses to both alcohol^31^^,^^32^^,^^36^ and various gut-brain peptides,^16^^,^^31^^,^^32^^,^^36^ as well as to consume high levels of alcohol that give pharmacologically relevant blood alcohol concentrations.^67^ Rats were also chosen for microdialysis experiments due to technical considerations with the injectable probe. For locomotor stimulation and CPP studies, male mice were used as they consistently display distinct stimulatory responses to alcohol, allowing comparison with existing data.^31^^,^^32^^,^^36^ While mice were not used for alcohol intake studies, similar outcomes are expected based on previous GLP-1R agonist studies across species.^14^^,^^34^^,^^36^ The combination of rat and mouse data is common in alcohol research, as each species can reflect different aspects of alcohol responses.^16^^,^^31^^,^^32^^,^^36^ We acknowledge the limitation of not including female mice in behavioural experiments, which may impact the generalisability of results across sexes. However, responses to acute stimulatory effects of alcohol and Ex4 are expected to be similar for both sexes. All animals were group-housed upon arrival and allowed to acclimatise for at least a week in rooms with standardised conditions (12/12-h light/dark cycle, 20 °C, and 50% humidity) with *ad libitum* access to regular chow (Teklad Rodent Diet, Envigo, Madison, WI, USA) and water. Animals were later single housed either following surgery or, in the case of alcohol intake studies, at the initiation of the alcohol intake baseline period to avoid damage to implants and/or to allow for individual intake measurements.

### Ethics

All experiments were approved by the Ethics Committee for Animal Research in Gothenburg, Sweden (ethical permits: 1457/18, 4685/23, 3348/20, and 3276/20), and followed the ARRIVE guidelines and 3Rs (refine, reduce, replace) principle. Total number of animals in each experiment and animals per group are listed in Table 1.

All animals used were handled on three occasions before the initiation of any experimental procedures and were only picked up supporting the body of the animal, without any tail-lifting. All behavioural and microdialysis experiments were conducted during the light phase, as stimulation is easier to observe at low activity. All animals were allowed to habituate to the testing room for 60 min before any experimental procedures were initiated. Alcohol intake studies were conducted during both the dark and light phases, with sessions starting at the beginning of the dark phase (animals were kept on a reversed 12/12-h light/dark cycle) as drinking behaviour is higher at this time point. Each animal was subjected to a single independent experiment. Animals were randomly allocated into balanced, equal-sized treatment groups for all behavioural and microdialysis studies and scored blindly as previously described,^36^ receiving only a single intra-LS injection. In the alcohol-drinking studies, each rat served as its own control in a counterbalanced within-subject design, with all drug treatments separated by 48 h, receiving a total of two intra-LS injections.

Although each specific experiment was conducted once, it involved multiple batches of testing per day and/or across several experimental days. This approach was necessary because, in most cases, only 4–6 animals could be tested simultaneously. Importantly, the observed results were consistent across all batches and days, suggesting that the effects were reliably replicated throughout the entire study.

Our pre-set exclusion criteria for removing rodents from the analysis included misplaced injection sites and probes, clogged guides, abnormal behaviours, poor health, or weight loss exceeding 15%. Technical difficulties in microdialysis and electrophysiology experiments, so that the experiments could not be conducted, were also considered as exclusion criteria when appropriate. Excluded number of animals and reason are listed in Table 1.

### General experimental procedures

**Fig. 1:**
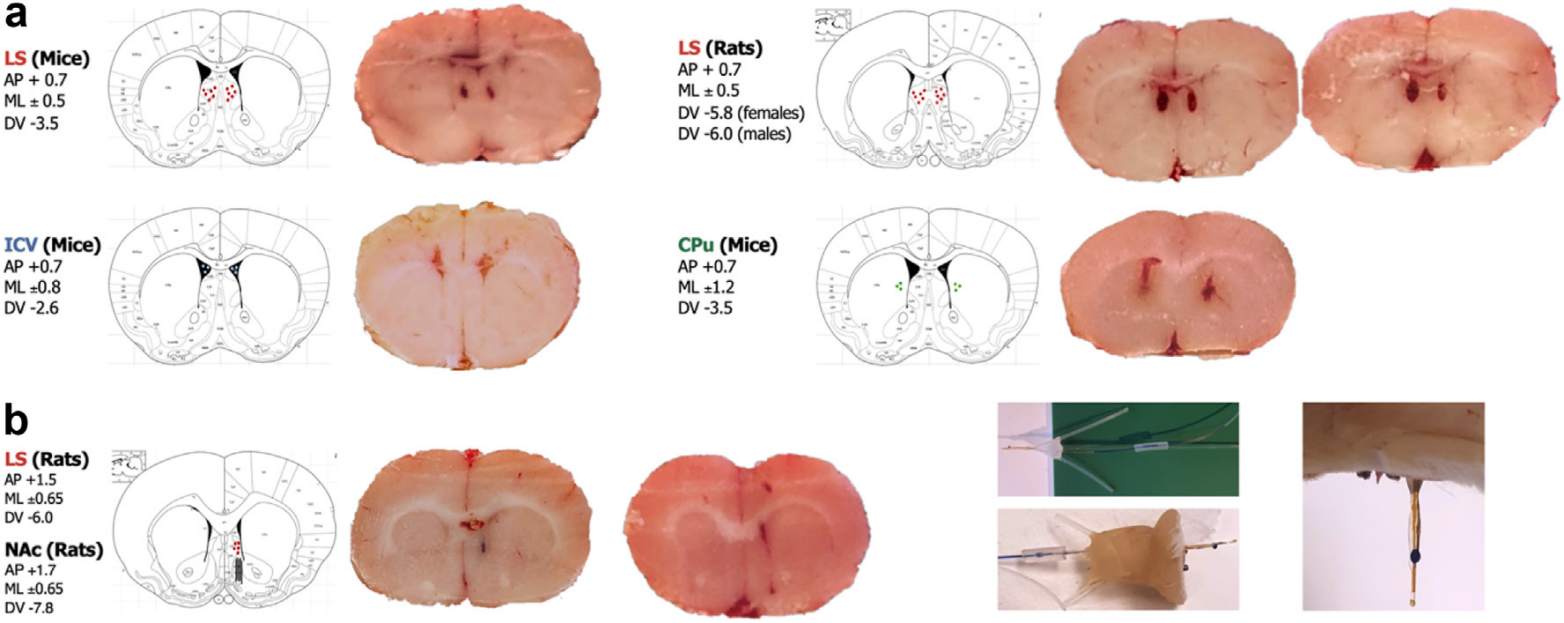
**Injection and probe placements. a.** Schematic representation of bilateral infusion sites within the lateral septum (LS), ventricles (ICV) and caudate putamen (CPu), including stereotaxic coordinates and examples of injection placements. **b.** Illustration of injectable probe placements using Pontamine sky blue ink (0.5 μL) (implanted with a 10° anterior-posterior angle), highlighting the infusion site within the LS and sampling site within the nucleus accumbens (NAc) shell.

To enable local microinfusion of drugs of interest into the LS, or the control region caudate putamen (CPu) which has lower abundance of GLP-1R,^18^^,^^68^ or lateral ventricles (ICV), bilateral guides or, for the microdialysis experiment, a unilateral (alternating left or right hemisphere) injectable probe (LS/NAc shell) were inserted four days before the experiments (Fig. 1a), as previously described.^31^^,^^69^ The coordinates for the LS, ICV, and CPu of mice and rats are shown in Fig. 1a. In brief, the animal was first anaesthetised with isoflurane (Isofluran Baxter, Apoteket AB, Gothenburg, Sweden, catalogue # 000890) using a Univentor 400 Anaesthesia Unit (Univentor Ltd., Zejtun, Malta). It was then placed in a stereotaxic frame (David Kopf Instruments; Tujunga, CA, USA) and kept on a heating pad to prevent hypothermia. Xylocaine with adrenaline (10 mg/ml, 5 μg/ml; Pfizer Inc., Apoteket AB, Gothenburg, Sweden, catalogue # 575593) was used as a local anaesthetic. Carprofen (Rimadyl®, 5 mg/kg, AstraZeneca; Apoteket AB, catalogue # 014920), 0.9% NaCl, and Viscotears were administered to relieve pain, rehydrate, and protect the eyes, respectively. The skull bone was exposed, and holes were drilled for the probe, guides (stainless steel, length 10 mm, with an o.d./i.d. of 0.6/0.45 mm), and anchoring screws. The probe or guides were secured to the skull bone with dental cement (DENTALON® Plus; Agntho's AB, Lidingö, Sweden). All animals were allowed to recover for four days before any experiments were initiated.

On the experimental day, 1 h before the microinfusion, dummy cannulas were inserted into the guides to full depth and then retracted to prevent spreading depression and to remove clotted blood.^31^^,^^69^ All microinfusions were given at a volume of 0.5 μL/side over 60 s, with the cannulas left in place for an additional 60 s to allow the drug to diffuse completely before being retracted. Following the conclusion of each experiment, brains were extracted and fixed. The location of the injection sites and/or microdialysis probe placement were verified histologically using a brain atlas (Paxinos et al., 2014), as done before.^31^^,^^69^ Only data from animals with the correct placement and those without brain haemorrhage were included in the statistical analysis (Fig. 1a–b).

### Drugs

**Fig. 2:**
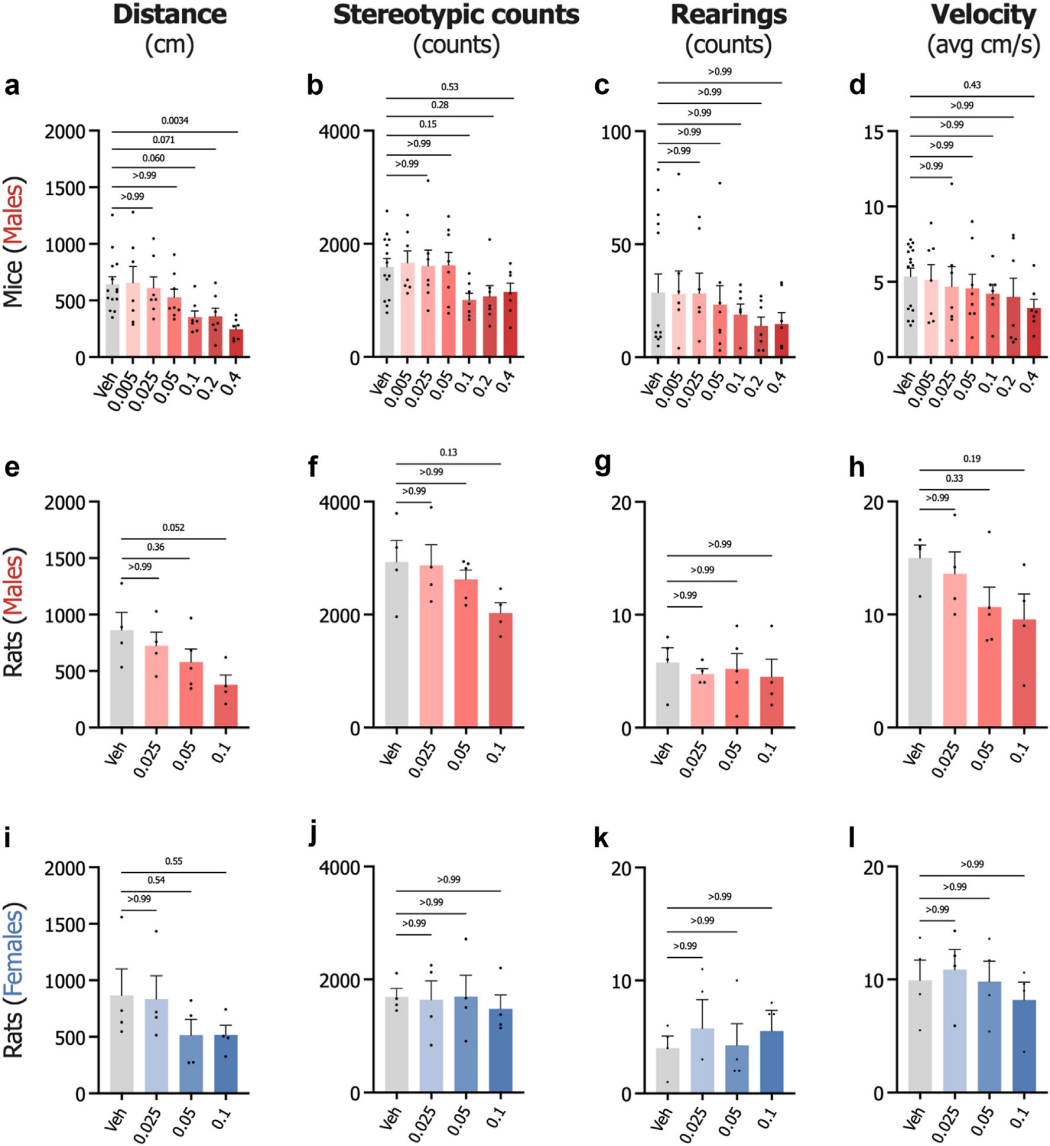
**Dose–response effects by infusion of exendin-4 into lateral septum on locomotor activity in mice and rats. a.** Examination of the effects of infusion of exendin-4 (Ex4) into lateral septum (LS) on locomotion in male mice reveals that higher doses (0.1, 0.2, and 0.4 μg), but not lower doses (0.005, 0.025, and 0.05 μg), reduced or tended to reduce locomotor activity compared to the vehicle (Veh) control (n = 7–14, F_6,50_ = 3.95, P = 0.0026, one-way ANOVA). **b–d.** Corresponding effects were observed in stereotypic counts, rearings, and velocity (n = 7–14, one-way ANOVA). **e.** In male rats, only the highest dose of Ex4 (0.1 μg) tended to affect locomotion, while lower doses (0.025 and 0.05 μg) were not statistically different compared to the vehicle (n = 4–5, F_3,13_ = 2.74, P = 0.086, one-way ANOVA). **f–h.** Similar dose-dependent effects were observed for stereotypic counts, rearings, and velocity (n = 4–5, one-way ANOVA) in male rats. **i.** In female rats, none of the administered doses of Ex4 (0.025, 0.05, and 0.1 μg) produced a statistically significant difference in locomotion compared to the vehicle control (n = 4, F_3,12_ = 1.22, P = 0.34, one-way ANOVA). **j–l**. Consistent with locomotion, no statistical differences were detected in stereotypic counts, rearings, and velocity (n = 4, one-way ANOVA) in female rats. Data are shown as individual data points with mean ± SEM presented.

As described before,^31^^,^^32^^,^^69^ for the behavioural and neurochemical experiments, alcohol (95% Ethanol, Solveco; Stockholm, Sweden, catalogue # 1000) was diluted with vehicle (0.9% NaCl) to a 15% (w/v) solution and administered intraperitoneally (IP) at a dose of 1.75 g/kg, 5 min prior to testing. In the alcohol intake studies, alcohol was diluted to a 20% (v/v) solution using tap water. For local administration in the brain, the GLP-1R agonist Ex4 and antagonist Exendin-3 (9-39) (Ex9) (Tocris Bioscience, Abingdon, UK, catalogue # 1933 and 2081) were diluted in vehicle (modified Ringer’s solution: 140 mM NaCl, 1.2 mM CaCl_2_, 3.0 mM KCl, and 1.0 mM MgCl_2_, Sigma–Aldrich, Darmstadt, Germany, catalogue #S9625, 383147, P9541, M8266) and administered 20 min before testing, Ex4 IP injection, or alcohol exposure. Ex4 was chosen for our experiments due to its established efficacy in central nervous system microinjection studies, particularly for investigating GLP-1R function in specific brain regions.^31^^,^^69^ While newer agonists like liraglutide and semaglutide show promise, Ex4's extensive use in rodent brain studies allows for direct comparisons with existing literature, enhancing the interpretability of our findings. The doses of Ex4 (0.025 μg, 0.0125 μg/side) and (0.05 μg, 0.025 μg/side) for both mice and rats were selected based on initial dose–response studies (Fig. 2a–l), where neither dose affected baseline locomotor activity or gross behaviour *per se*. The dose of Ex9 (10 μg, 5 μg/side) used in this study was based on previous studies focusing on reward-related responses and local brain-site inhibition of GLP-1R signalling.^27^^,^^55^^,^^56^^,^^69^^,^^70^ It should however be noted, as a limitation, that no dose–response study or test of drug effects in adjacent areas for Ex9 were performed in this project. In one locomotor activity experiment, Ex4 was injected intraperitoneally (IP) at a dose of 2.4 μg/kg (diluted in 0.9% NaCl) 20 min before alcohol administration, as before.^69^

For the electrophysiology experiments, the concentrations of Ex4 (500 nM) and the GABA_A_ receptor (GABA_A_R) antagonist bicuculline-methiodide (Bic, 20 μM, Sigma–Aldrich; Darmstadt, Germany, catalogue # 14343) were based on previous studies as these concentrations produce robust effects in *ex vivo* electrophysiology^24^^,^^25^^,^^28^^,^^71^, ^72^, ^73^ and are around the same range as our rat *in vivo* Ex4 doses (0.025–0.05 μg/6–12 μM). It should however be noted, as a limitation, that only one concentration of Ex4 and Bic were tested in this project. Both compounds were diluted in artificial cerebrospinal fluid (aCSF; 124 mM NaCl, 4.5 mM KCl, 2 mM CaCl_2_, 1 mM MgCl_2_, 26 mM NaHCO_3_, 1.2 mM NaH_2_PO_4_, and 10 mM D-glucose, with a sucrose-adjusted osmolarity of 315–320 mOsm, Sigma–Aldrich).

### Locomotor activity

Horizontal and vertical activity were recorded in six sound-attenuated, ventilated, and dimly lit (3 lx) locomotor activity chambers (42 × 42 × 20 cm; Open Field Activity System, Med Associates Inc., Georgia, VT, USA). Animal movement was detected in a two-layered grid of infrared photobeams and registered using the Activity Monitor software (Version 7, Med Associates Inc., Georgia, VT, USA). All animals were allowed to habituate to the test arena for 60 min before drug administration. Activity registration was started 5 min after the last injection and recorded for 60 min.

### Conditioned place preference

Expression of CPP was conducted as previously described^31^^,^^32^^,^^69^ in four two-chambered arenas (50 × 24 × 24 cm, custom-made at the University of Gothenburg, Sweden), dimly lit (3 lx). The two chambers are defined using different tactile and visual cues. In brief, the initial place preference was assessed by placing mice in the centre of the CPP arena and allowing 20 min of free access to both chambers during the pre-test (day 1). Conditioning sessions (day 2–5, 20 min each) followed a biased design, pairing alcohol with the least preferred chamber and vehicle with the preferred one. Each day, mice received one alcohol and one vehicle injection in a balanced design, alternating between morning and afternoon. On the test day (day 6), mice were bilaterally injected with either Ex4 or vehicle into the LS before being placed at the midline of the CPP box for a 20-min test of free access to both chambers. An additional control experiment was conducted to assess the effect of Ex4 in the LS on CPP *per se*, independently of alcohol. In this experiment, mice underwent the same procedure but received vehicle injections in both chambers during conditioning. Experiments were analysed with Observer XT software version 15 (Noldus, Wagenegen, the Netherlands). The expression of CPP was calculated as the difference in the percentage of total time spent in the drug-paired compartment during the pre-test and test sessions.

### Microdialysis and monoamine analysis

Owing to the close proximity of the LS and NAc shell brain regions, an I-shaped injectable microdialysis probe was constructed (Fig. 1b), adapted from.^74^ This design enabled local microinfusion of vehicle or Ex4 into the LS while simultaneously sampling in the NAc shell. The probe (dialysis membrane; 20 kDa cut off with a 2 mm exposed length, HOSPAL, Gambro, Lund, Sweden) was surgically implanted four days before the experiment. The placement of the injection was confirmed *post-mortem* with Pontamine sky blue ink (0.5 μL) (Fig. 1b), as previously described.^32^^,^^33^^,^^69^

On the day of the experiment, the probe was connected to a pump and perfused with Ringer’s solution at a rate of 1.6 μl/min. A 2-h equilibrium period was allowed before the initiation of the experiment. Samples were collected every 20 min throughout the experiment. Following a baseline period (minute −40 to 0), Ex4 or vehicle was infused at min 0. Twenty minutes later, alcohol or vehicle was injected (minute 20), and nine additional samples were collected (minute 40–200).

Collected microdialysate samples were quantified using high-performance liquid chromatography (HPLC) followed by electrochemical detection. The analytical methods for monoamine neurotransmitters (noradrenaline (NA), dopamine (DA), and serotonin (5-HT), along with their metabolites 3,4-dihydroxyphenylacetic acid (DOPAC), 3-methoxy-4-hydroxyphenethylamine (3-MT), homovanillic acid (HVA), normetanephrine (NM), 5-hydroxyindoleacetic acid (5-HIAA), and the dopamine precursor L-β-3,4-dihydroxyphenylalanine (L-DOPA), were inspired by,^75^ utilised two isocratic chromatographic separations dedicated to amines or acids.

The amines (NA, DA, NM, 3-MT, and 5-HT) were separated using two-dimensional reverse-phase ion-pair HPLC on two fully porous silica C18 reverse-phase columns (Luna C18 (2), 20 × 2 mm and 50 × 2 mm, 3 μm particle size, 100 Å pore size, Phenomenex, Værløse, Denmark). The two mobile phases consisted of 86% (v/v) of a buffered aqueous solution (5 mM citric acid monohydrate, 10 mM di-sodium citrate trihydrate, 0.1 mM Na_2_-EDTA, with 0.7 mM sodium-1-dodecanesulfonate additionally added to the second mobile phase, pH 5.5), 10% (v/v) acetonitrile, and 4% (v/v) tetrahydrofuran, delivered at a flow rates of 0.300 ml/min, applied potential for the detector was 400 mW.

The acids (L-DOPA, DOPAC, 5-HIAA, and HVA) were separated using reverse-phase ion-pair HPLC on the same type of column (Luna C18 (2), 50 × 2 mm). The mobile phase consisted of 92% (v/v) of a buffered aqueous solution (53.3 mM citric acid monohydrate, 13.2 mM di-potassium hydrogen phosphate trihydrate, 0.1 mM Na_2_-EDTA, 0.2 mM sodium-1-dodecanesulfonate, pH adjusted to 2.95 with 5 M NaOH), and 8% (v/v) methanol, delivered at a flow rate of 0.300 ml/min and an applied potential of 600 mW.

Changes in monoamine levels were calculated as a percentage of the mean of the three baseline values before Ex4/vehicle treatment. Additionally, the area under the curve (AUC) following the last injection (minutes 20–200) was calculated.

### Intermittent access two-bottle choice—alcohol intake studies

This alcohol-drinking paradigm was conducted as before.^31^^,^^32^^,^^69^ Rats had access to alcohol and water during three 24-h sessions per week (Monday, Wednesday, and Friday). Only water was provided on the other days, bottles were always swapped at the onset of the dark phase. Rats were exposed to alcohol for 8–10 weeks (baseline period) before the initiation of the experiments. During this baseline period, the intake of alcohol, water, and food was measured daily, and body weight was recorded weekly. It is commonly observed, both in our and other studies,^34^^,^^36^^,^^76^^,^^77^ that female rodents consume more alcohol than males. On each treatment day, following LS microinfusion of Ex4/Ex9/vehicle, the cumulative intake of alcohol, food, and water was measured 2, 4, and 24 h after alcohol access. The changes in body weight were recorded after 24 h.

### Quantitative polymerase chain reaction (qPCR)–gene expression

Gene expression analysis of GLP-1R in treatment-naïve alcohol-consuming (8–10 weeks) rats was conducted as previously described.^31^^,^^78^ In brief, rats were given access to alcohol for 24 h before being euthanised at the onset of the dark phase. The brains were extracted and placed in a brain-slicing matrix, and the LS as well as the positive control areas NAc, VTA, and LDTg,^31^ were punched out. These tissue samples were immediately frozen on dry ice and stored at −80 °C until further analysis. The total RNA was extracted, purified, and amplified, with all samples run in triplicates. The expression of the *glp-1r gene* (ThermoFisher, Rn00562406_m1) was normalised to the geometric mean of *β-actin* (ThermoFisher, Rn00667869_m1). The comparative threshold cycle (Ct) method was used to analyse the qPCR data.^79^ Low alcohol-consuming rats of each sex were used as the control group. The data are presented as relative gene expression of the GLP-1R in high versus low alcohol-consuming animals in the form of 2^−DDCt^, or presented as delta threshold cycle (DCt) values.

### Electrophysiology—field potential recordings

*Ex vivo* field potential recordings of brain slices from treatment-naive, alcohol-consuming rats (8–10 weeks, n = 10 per sex, with 24 h alcohol access before euthanisation at the onset of the dark phase) were conducted as previously described.^73^^,^^80^ In brief, brains were removed and submerged in ice-cold modified artificial cerebrospinal fluid (aCSF) cutting solution (220 mM sucrose, 2 mM KCl, 6 mM MgCl_2_, 26 mM NaHCO_3_, 1.3 mM NaH_2_PO_4_, 0.2 mM CaCl_2_, and 10 mM D-glucose; Sigma–Aldrich, Darmstadt, Germany), continuously bubbled with a gas mixture of 95% O_2_/5% CO_2_. Brains were sectioned coronally at 300 μm using a vibratome to obtain slices containing the LS region. The slices were transferred to a custom-made incubation chamber with aCSF and continuously bubbled with 95% O_2_/5% CO_2_. The slices were incubated in aCSF for 30 min at 30 °C and then at room temperature for the remainder of the day.

Slices were then placed in the recording chambers for field-potential recordings in the LS. A stimulation electrode was positioned locally, 0.2–0.3 mm from the recording electrode (resistance 2.5–4.5 MΩ) was positioned locally, and the amplitude of evoked population spike was measured. Population spike (PS)s was evoked with a stimulation frequency of 0.05 Hz, and the stimulus intensity was set to yield a population spike amplitude of approximately half the maximal evoked response. Stimulus-response curves were first assessed by a stepwise increase in stimulation intensity. Following a stable baseline in aCSF for 10 min, drug perfusion (Ex4/Bic/aCSF) was initiated, and the effects on the population spike amplitude were investigated. To assess changes in release probability after drug treatment, a paired-pulse ratio (PPR) stimulation protocol (0.1 Hz, 50 ms interpulse interval) was used.

PPR was calculated by dividing the second PS amplitude by the first evoked response. Data were acquired using Clampfit 10.2 (Molecular Devices, Foster City, CA, USA).

### Statistics

To adhere to the 3R the number of animals per treatment group was minimised based on prior experience. The sample size was not calculated using unstandardised association measure values, and the actual power of the study might not match expectations and may thus be underpowered. However, the sample size was calculated as done before using a 5% significance level, an effect size of at least 0.2 standard deviations, a two-tailed hypothesis, and 80% study power, and are in line with previous studies and matches the expected treatment outcome.^31^^,^^36^^,^^81^ A minimum of seven animals was considered sufficient to detect statistically significant effects. In certain dose–response and locomotor activity control experiments, fewer animals were used, as these studies aimed to confirm dose-range appropriateness and assess potential effects following injections into LS-adjacent regions.

Statistical analyses were conducted using GraphPad Prism (version 10.3.0, GraphPad Software Inc., Boston, MA, USA). Normal distribution was tested using the Shapiro–Wilk test and by visual inspection of the generated Q-Q-plot. As these tests showed normal distribution parametric tests were used. All tests were two-tailed, with an alpha level of 0.05. Paired or unpaired student’s t-tests were used for comparisons between two groups. When comparing three or more groups, one-way analysis of variance (ANOVA) with Bonferroni post-hoc test was used. Homogeneity of variance were assessed using the F-test (unpaired t-test) or Brown–Forsythe test (one-way ANOVA). For the microdialysis and electrophysiology experiments, repeated-measures two-way ANOVA (factors treatment and time) was performed, with a Greenhouse-Geisser correction applied to adjust for lack of sphericity when appropriate, followed by Bonferroni post-hoc tests for comparisons between different treatments and at specific time points. Correlation analyses were performed using Pearson’s correlation. Data are presented as individual data points with mean ± standard error of the mean (SEM) displayed, unless otherwise stated.

### Role of funders

The funding sources did not play a role for the study design, collection, analysis, and interpretation.

## Results

### LS GLP-1R is involved in mediating the systemic effect of Ex4 in male mice

**Fig. 3:**
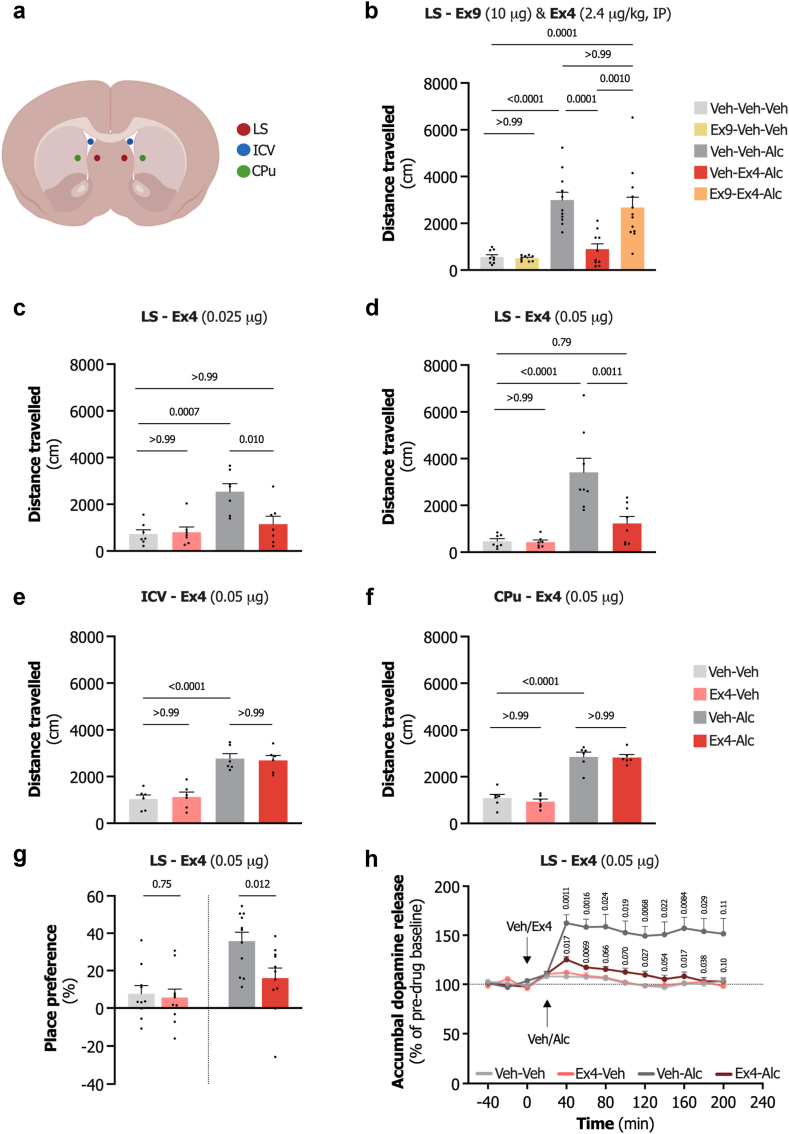
**Impact of lateral septum GLP-1 receptor signalling on alcohol-related reward responses in male rodents. a.** Schematic depiction of the injection sites. **b.** Intra-lateral septum (LS) administration of the GLP-1 receptor antagonist exendin-3 (9-39) (Ex9, 10 μg) blocks the ability of systemic exendin-4 (Ex4, 2.4 μg/kg, IP) to attenuate alcohol (Alc, 1.75 g/kg, IP)-induced locomotor activity in male mice (n = 9–12, one-way ANOVA). **c.** Alcohol (1.75 g/kg, IP)-induced locomotor stimulation in male mice is inhibited by LS infusion of 0.025 μg Ex4 (n = 7, one-way ANOVA). **d.** LS infusion of 0.05 μg Ex4 similarly blocks alcohol (1.75 g/kg, IP)-induced locomotor stimulation in male mice (n = 7–8, one-way ANOVA). **e.** There is no statistically significant effect by intracerebroventricular (ICV) infusion of 0.05 μg Ex4 on alcohol (1.75 g/kg, IP)-induced locomotor stimulation in male mice (n = 6, one-way ANOVA). **f.** Alcohol (1.75 g/kg, IP)-induced locomotor stimulation remains statistically unaffected by caudate putamen (CPu) infusion of 0.05 μg Ex4 in male mice (n = 6, one-way ANOVA). **g.** Intra-LS administration of Ex4 (0.05 μg) in male mice reduces alcohol (1.75 g/kg, IP)-induced conditioned place preference (CPP) without statistically different altering CPP by itself (n = 10–11, unpaired t-tests). **h.** LS infusion of Ex4 (0.05 μg) attenuates alcohol (1.75 g/kg, IP)-evoked dopamine release in the nucleus accumbens (NAc) shell in male rats (n = 8 per group, repeated measures two-way ANOVA). Data are shown as individual data points with mean ± SEM presented. The animation was created using BioRender.

Systemic GLP-1R agonist Ex4 has previously been demonstrated to attenuate alcohol-induced locomotion.^32^ In this study, we evaluated the role of LS GLP-1R in the systemic effects of Ex4. The mice were first treated with GLP-1R antagonist Ex9 (10 μg) or vehicle in the LS. Thereafter, they received a systemic injection of Ex4 (2.4 μg/kg, IP) or vehicle, followed by a systemic administration of alcohol (1.75 g/kg, IP) or vehicle. As shown in Fig. 3b (F_4,46_ = 15.84, P < 0.0001, one-way ANOVA), alcohol caused locomotor stimulation (P < 0.0001) compared to vehicle. Systemic administration of Ex4 blocked this effect (P = 0.0001). However, in mice treated with intra-LS Ex9 before systemic Ex4 administration alcohol-induced locomotion was observed at a level similar to that observed in alcohol-treated mice (P > 0.99). Additionally, intra-LS Ex9 did not statistically differ in locomotion (P > 0.99) compared to vehicle.

### Intra-LS Ex4 dose–response studies in mice and rats

The Ex4 dose–response study in male mice (Fig. 2a) revealed dose-dependent reductions in locomotor activity for the three highest doses compared to vehicle. The 0.1 μg and 0.2 μg doses reduced distance travelled by 285.3 ± 62.6 cm and 244.2 ± 66.6 cm, respectively. The 0.4 μg dose produced the largest effect, with a reduction of 423.9 ± 32.4 cm in locomotion. Similar trends were observed in additional behavioural parameters (Fig. 2b–d). Based on these results, we selected the 0.025 and 0.05 μg doses for subsequent experiments in mice, as the mean differences for these doses were less than 150 cm compared to vehicle, indicating minimal impact on general locomotor activity.

In male rats (Fig. 2e), the 0.025 μg dose reduced locomotion by 137.6 ± 62.6 cm compared to vehicle, while the 0.1 μg dose reduced locomotion by 483.0 ± 86.8 cm. Similar effects were noted in additional behavioural parameters (Fig. 2f–h). Female rats (Fig. 2i) showed that the 0.025 μg dose reduced locomotion by 32.0 ± 204.7 cm compared to vehicle, while the 0.1 μg dose reduced locomotion by 351.0 ± 86.0 cm. Additional behavioural parameters showed similar trends (Fig. 2j–l). Guided by the principle of minimising effects on general locomotion, as with the mice, we chose the 0.025 μg dose for both sexes in the initial alcohol drinking studies.

### LS, but not ICV or CPu, Ex4 infusion mitigates alcohol-induced locomotor stimulation in male mice

In these experiments, the effects of bilateral infusions of Ex4 at doses of 0.025 or 0.05 μg into the LS, ICV, or CPu on alcohol (1.75 g/kg, IP)-induced locomotion in male mice were evaluated.

The first experiment explored the effect of 0.025 μg Ex4 into the LS. Alcohol caused significant locomotor stimulation (P = 0.0007) compared to vehicle, which was effectively blocked by Ex4 (P = 0.010) (F_3,24_ = 9.24, P = 0.0003, one-way ANOVA; Fig. 3c). No statistical differences were observed between the vehicle–vehicle and Ex4-alcohol groups (P > 0.99). This result was corroborated by a subsequent experiment that used a higher dose of Ex4 (0.05 μg). Again, alcohol-induced hyperlocomotion (P < 0.0001) compared to vehicle. This was attenuated in the Ex4-treated group (P = 0.0011), with no statistical differences between the vehicle–vehicle and Ex4-alcohol treated animals (P = 0.92) (F_3,26_ = 15.11, P < 0.0001, one-way ANOVA; Fig. 3d).

Further experiments were conducted to ensure that the observed effects were localised to the LS and not due to diffusion to the adjacent areas. In these experiments, Ex4 (0.05 μg) was infused either ICV or into the more laterally located CPu. While a clear alcohol response was observed in the ICV experiment (P < 0.0001) Ex4 had no statistically observable effect on alcohol-induced locomotor activity (P > 0.99) (F_3,20_ = 22.53, P < 0.0001, one-way ANOVA; Fig. 3e). Alcohol-elicited locomotor stimulation was also visible in the CPu experiment (P < 0.0001), intra CPu-Ex4 had no statistically observable effect on alcohol-induced locomotor activity (P > 0.99) (F_3,20_ = 47.71, P < 0.0001, one-way ANOVA; Fig. 3f).

In neither of these four locomotor activity experiments, local Ex4 infusion affected locomotion *per se* (P > 0.99; Fig. 3c–f).

### Activation of LS GLP-1R attenuates the rewarding effects of alcohol in male rodents

To better understand the role of LS GLP-1R activation in alcohol reward-related behaviour, we next evaluated how Ex4 (0.05 μg) infusion into the LS affected the expression CPP in male mice. In the first experiment, a control experiment, vehicle was paired with both sides of the apparatus to evaluate if intra-LS Ex4 effect the place conditioning in itself. The results indicated no statistically significant influence of Ex4 on CPP (t_18_ = 0.32, P = 0.75, unpaired t-test; Fig. 3g). In the subsequent experiment, a significant preference for the alcohol (1.75 g/kg, IP)-paired environment was observed in the vehicle group, but significantly attenuated in the intra-LS Ex4 treated group (t_20_ = 2.75, P = 0.012, unpaired t-test; Fig. 3g).

To further elucidate the role of LS GLP-1R in regulating the rewarding effects of alcohol, microdialysis was employed in male rats to measure the accumbal monoamine release following alcohol (1.75 g/kg, IP) administration (Fig. 3h). Alcohol administration resulted in a pronounced increase in dopamine release compared to vehicle (treatment F_3,28_ = 22.76, P < 0.001, interaction F_29,348_ = 9.29, P < 0.01 at time points 40, 60, 120, 160, and P < 0.05, at time points 80, 100, 140, and 180, repeated-measures two-way ANOVA). This increase in dopamine was significantly attenuated by intra-LS Ex4 (0.05 μg) administration (P < 0.05, at time points 40, 120, 160, and 180, and P < 0.01 at time point 60). The AUC analysis revealed that local LS Ex4 infusion in itself did not statistically effect dopamine release (P > 0.99, one-way ANOVA), neither was there any statistical differences between vehicle–vehicle and Ex4-alcohol treated animals (P = 0.89). Similar effects were observed for the dopamine metabolites DOPAC (treatment F_3,28_ = 4.41, P = 0.012, interaction F_28,336_ = 14.57, P < 0.0001, repeated-measures two-way ANOVA), 3-MT (treatment F_3,28_ = 6.83, P = 0.0013, interaction F_28,336_ = 4.37, P < 0.0001, repeated-measures two-way ANOVA), and HVA (treatment F_3,28_ = 6.65, P = 0.0016, interaction F_28,336_ = 14.24, P < 0.0001, repeated-measures two-way ANOVA) Supplementary Fig. S1a–c, in which intra-LS Ex4 significantly mitigated the alcohol-induced increase. No statistical differences were observed across treatment groups for the remaining monoamines (Supplementary Fig. S1d–h). A summary of the AUC analyses for all measured monoamines is provided in Supplementary Fig. S1i.

### LS GLP-1R activation dose-dependently reduces alcohol intake in rats of both sexes

A series of studies were conducted to investigate the role of LS GLP-1R activation in voluntary alcohol consumption in male and female rats. In each study, rats received an LS infusion of Ex4 (0.025 μg or 0.05 μg) or vehicle, followed by 24 h access to alcohol, water, and food. A counterbalanced within-subject design was used in these studies.

**Fig. 4:**
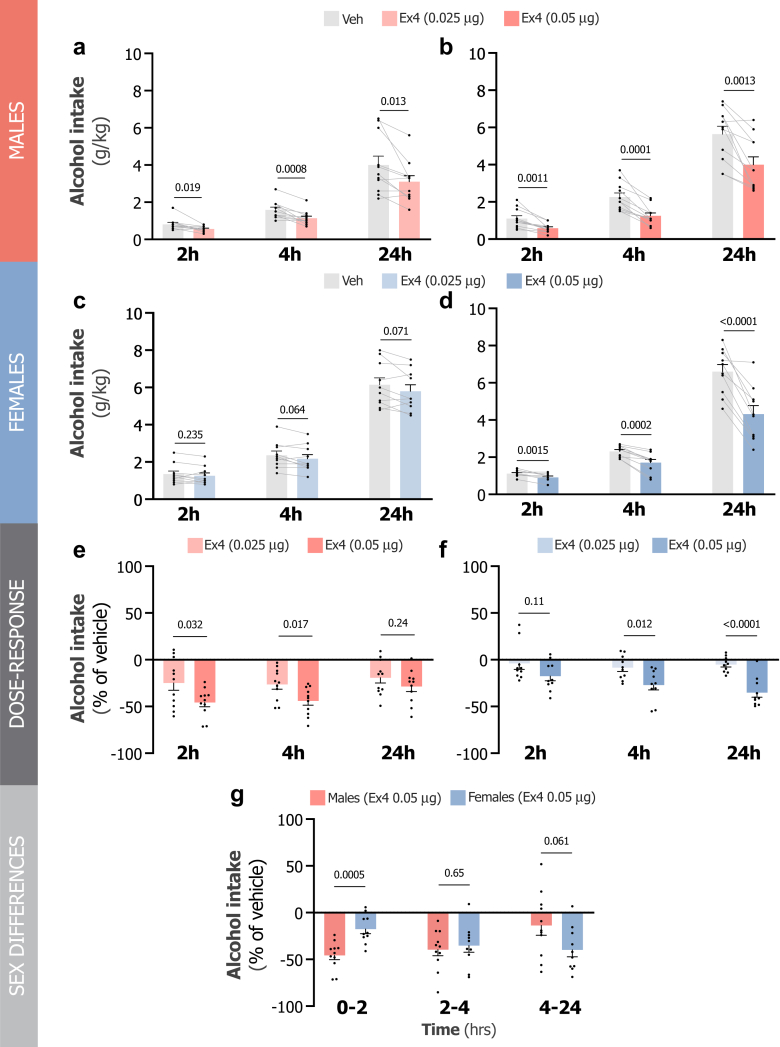
**Activation of lateral septum GLP-1 receptor reduces alcohol intake in rats of both sexes. a.** Intra-lateral septum (LS) infusion of 0.025 μg exendin-4 (Ex4) significantly reduces alcohol intake at 2, 4, and 24 h compared to vehicle in male rats (n = 11, paired t-test). **b.** Alcohol intake at 2, 4, and 24 h is further decreased by LS infusion of 0.05 μg Ex4 compared to vehicle in male rats (n = 11, paired t-test). **c.** Intra-LS infusion of 0.025 μg Ex4 tends to reduce alcohol intake at 2, 4, and 24 h compared to vehicle in female rats (n = 10, paired t-test). **d.** Alcohol intake at 2, 4, and 24 h is significantly decreased by LS infusion of 0.05 μg Ex4 compared to vehicle in female rats (n = 10, paired t-test). **e.** A dose–response relationship is observed for Ex4 in the reduction of alcohol intake in male rats (n = 11, unpaired t-test). **f.** A dose–response relationship is also observed in female rats, where Ex4 influences alcohol intake (n = 10, unpaired t-test). **g.** A sex-dependent difference is evident in the onset of Ex4's effects on alcohol intake (n = 10–11, unpaired t-test). Data are shown as individual data points with mean ± SEM presented.

In the first study, the effects of a low Ex4 dose (0.025 μg) were evaluated in male rats. As shown in Fig. 4a, Ex4 significantly reduced alcohol intake at 2 h (t_10_ = 2.80, P = 0.019), 4 h (t_10_ = 4.75, P = 0.0008), and 24 h (t_10_ = 3.03, P = 0.013, paired t-test) compared to vehicle. In the second study, a higher Ex4 dose (0.05 μg) was tested in male rats. Again, Ex4 significantly decreased alcohol intake at 2 h (t_10_ = 4.50, P = 0.0011), 4 h (t_10_ = 6.12, P = 0.0001), and 24 h (t_10_ = 4.41, P = 0.0013, paired t-test) relative to the vehicle group (Fig. 4b). The third study examined the effects of a low Ex4 dose (0.025 μg) on alcohol intake in female rats. As shown in Fig. 4c, Ex4 did not statistically reduce alcohol intake. However, there were observable trends towards a reduction at 4 h (t_9_ = 2.11, P = 0.064) and 24 h (t_9_ = 2.05, P = 0.071, paired t-test) compared to vehicle. In the final experiment, a higher Ex4 dose (0.05 μg) was administered to LS of female rats. This higher dose of Ex4 significantly reduced alcohol intake at 2 h (t_9_ = 4.47, P = 0.0015), 4 h (t_9_ = 6.00, P = 0.0002), and 24 h (t_9_ = 6.85, P < 0.0001, paired t-test) compared to that in the vehicle group (Fig. 4d).

The higher dose of Ex4 caused a more pronounced reduction in alcohol consumption than the lower dose in both male and female rats, indicating a dose–response relationship. As shown in Fig. 4e, the effects were most pronounced at 2 h (t_20_ = 2.31, P = 0.032) and 4 h (t_20_ = 2.60, P = 0.017, unpaired t-test) in male rats, whereas they were greater at 4 h (t_18_ = 2.79, P = 0.012) and 24 h (t_18_ = 5.46, P < 0.0001, unpaired t-test; Fig. 4f) in female rats. This dose–response relationship was not observed for the other parameters (data not shown). Notably, the onset of higher dose effects differed between sexes, as shown in Fig. 4g. This is demonstrated as Ex4 exerted a significantly greater effect in male rats during the initial 2 h (t_19_ = 4.20, P = 0.0005, unpaired t-test) than in female rats, whereas in female rats, the strongest effect, although not statistically different, was more pronounced during the 4–24 h interval (t_19_ = 2.60, P = 0.061).

Additionally, the alcohol intake levels for the vehicle animals were at similar levels to baseline intake across all test days for both sexes in all four drinking studies (Supplementary Fig. S2a–d).

No statistical differences between treatments on food and water intake or body weight were observed in the first study in male rats (Supplementary Fig. S3a–c). As in the first study, no statistical differences between treatments on food or water intake were detected in the second study (Supplementary Fig. S3d and e); however, a reduction in body weight was observed (t_10_ = 2.24, P = 0.049, paired t-test; Supplementary Fig. S3f). In the third study in female rats, no statistical differences between treatments were noted on food and water intake or body weight (Supplementary Fig. S3g–i). In the final study, there were no statistical differences between treatments on food or water intake observed at 2 and 4 h (Supplementary Fig. S3j–k), although a trend towards reduced food intake at 24 h was noted (t_9_ = 2.09, P = 0.067, paired t-test; Supplementary Fig. S3j) and body weight was significantly decreased (t_9_ = 1.82, P = 0.012; Supplementary Fig. S3l).

### Alcohol-preferring male rats exhibit increased LS GLP-1R levels

Prolonged alcohol exposure has previously been shown to potentially alter the GLP-1 system.^31^^,^^82^, ^83^, ^84^, ^85^ To complement our behavioural findings, we next assessed if long-term alcohol consumption could affect central GLP-1R expression in reward-related brain regions of treatment-naive, alcohol-consuming male, and female rats. Rats were categorised as low- or high-consumers within each sex, based on their alcohol intake (cut-off >3.5 g/kg/d for males and >5.0 g/kg/d for females; Supplementary Fig. S4a and b). The DCt GLP-1R expression values were significant between sexes (t_46_ = 2.37, P = 0.022, unpaired t-test). An effect was also noticed in high alcohol-consuming males (t_22_ = 4.59, P = 0.0001, unpaired t-test) compared to the high consuming females (Supplementary Fig. S4c).

**Fig. 5:**
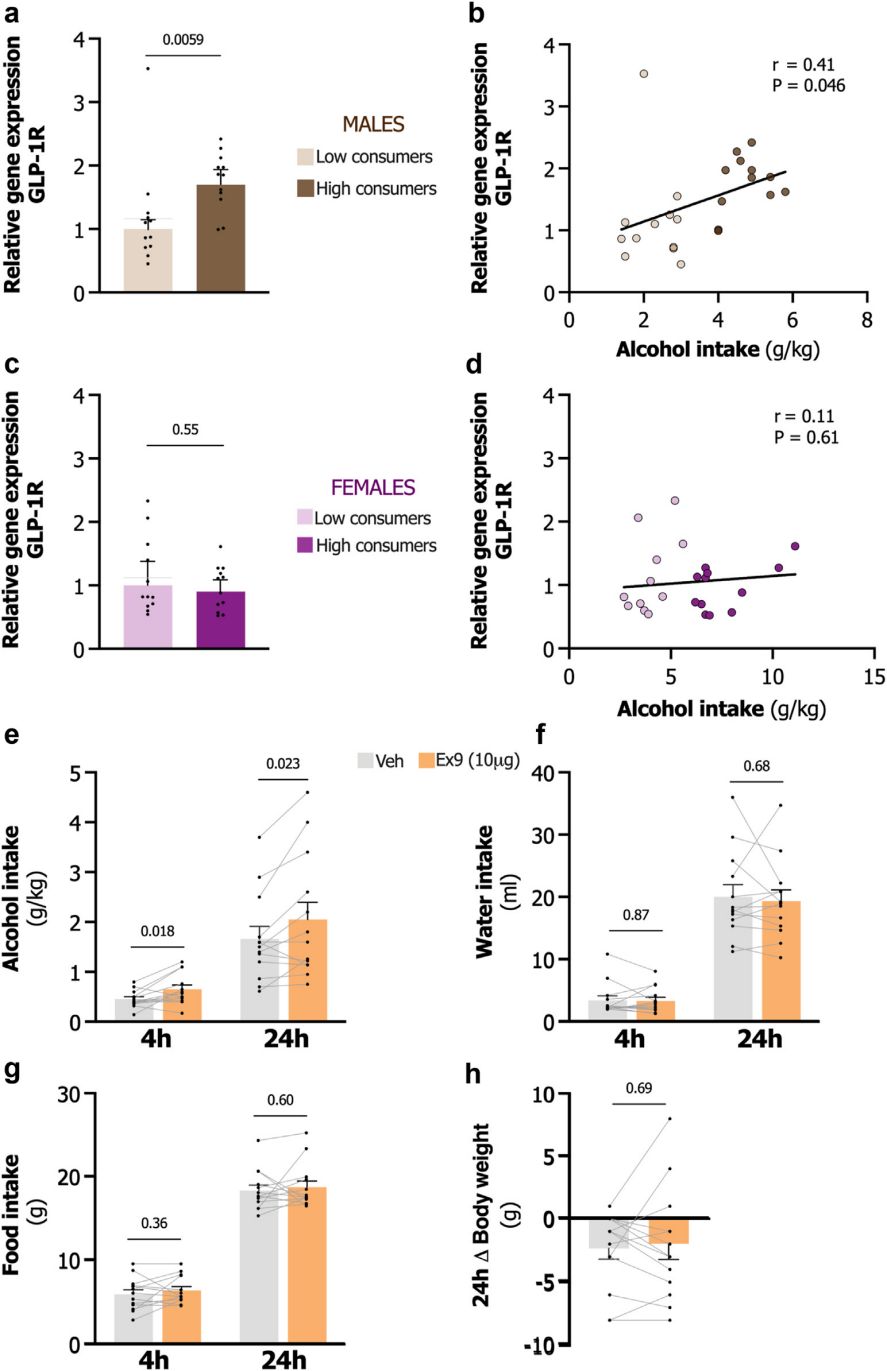
**Long-term alcohol consumption modifies lateral septum GLP-1 receptor expression in male, but not female rats and inhibition of these receptors promotes alcohol intake. a.** Lateral septum (LS) GLP-1 receptor (GLP-1R) expression is significantly elevated in high consuming compared to low alcohol-consuming male rats, shown as relative gene expression normalised to the low-consumers (n = 12, unpaired t-test of delta threshold cycle (DCt) values). **b.** A positive correlation between LS GLP-1R expression and alcohol intake is observed in male rats (n = 24, Pearson correlation test). **c.** No statistically significant change in LS GLP-1R expression is found between high and low alcohol-consuming female rats, shown as relative gene expression normalised to the low-consumers (n = 12, unpaired t-test of DCt values). **d.** LS GLP-1R expression does not statistically correlate with alcohol intake in female rats (n = 24, Pearson correlation test). **e.** Intra-LS infusion of 10 μg GLP-1R antagonist exendin-3 (9-39) (Ex9) significantly increases alcohol intake at 4 and 24 h post-infusion compared to vehicle in male rats (n = 13, paired t-test). **f.** Water intake at 4 (t_12_ = 0.16, P = 0.87) and 24 (t_12_ = 0.42, P = 0.68) hours does not show statistically significant changes following intra-LS infusion of 10 μg Ex9 compared to vehicle in male rats (n = 13, paired t-test). **g.** Food intake at 4 (t_12_ = 0.95, P = 0.36) and 24 (t_12_ = 0.53, P = 0.60) hours remains statistically unaffected by intra-LS infusion of 10 μg Ex9 compared to vehicle in male rats (n = 13, paired t-test). **h.** Body weight is not statistically altered by intra-LS infusion of 10 μg Ex9 compared to vehicle in male rats (n = 13, t_12_ = 0.41, P = 0.69, paired t-test). Data are shown as individual data points with mean ± SEM presented.

High levels of alcohol consumption were associated with increased LS GLP-1R expression in male rats (t_22_ = 3.05, P = 0.0059, unpaired t-test; Fig. 5a) compared to the animals that consumed low levels of alcohol. Additionally, a significant positive correlation was observed between GLP-1R expression in the LS and alcohol intake (r = 0.41; 95% CI; 0.0088–0.70), P = 0.046, Pearson’s correlation; Fig. 5b). Elevated GLP-1R expression was also noted in the NAc of male rats (t_22_ = 2.36, P = 0.028) but no statistical difference was observed in the VTA or LDTg (Supplementary Fig. S4d).

In contrast, high alcohol-consuming female rats demonstrated no statistical differences in GLP-1R expression in the LS (t_22_ = 0.61, P = 0.55, unpaired t-test; Fig. 5c) or any other brain region tested (Supplementary Fig. S4e) compared to the low alcohol-consuming rats. Furthermore, no statistically significant correlation was found between GLP-1R expression in the LS and alcohol intake in females (r = 0.11; 95% CI; −0.31-0.49), P = 0.61, Pearson’s correlation; Fig. 5d).

### Inhibition of LS GLP-1R promotes alcohol intake in male rats

Given the observed changes in GLP-1R expression in alcohol-consuming male rats, an additional alcohol-drinking experiment was conducted to explore whether inhibiting LS GLP-1R, simulating a loss-of-function, potentially could drive voluntary alcohol consumption. To reduce the number of animals used, female rats were not included in this experiment, but a similar outcome is expected in both sexes. Utilising a counterbalanced within-subjects design, rats received an LS infusion of Ex9 (10 μg) or vehicle, followed by 24 h access to alcohol, water, and food. As illustrated in Fig. 5e, Ex9 significantly increased alcohol intake at both 4 h (t_12_ = 2.75, P = 0.018) and 24 h (t_12_ = 2.60, P = 0.023, paired t-test) compared to vehicle. No statistical differences were observed on any of the other parameters (Fig. 5f–h).

### GLP-1R activation suppresses LS *ex vivo* neurotransmission in brain slices from alcohol-consuming male and female rats

To gain deeper mechanistic insights into the neurobiological mechanisms underlying LS GLP-1R mediated inhibition of reward-related processes, *ex vivo* field potential recordings were conducted in brain slices from treatment-naive, alcohol-consuming male and female rats.

**Fig. 6:**
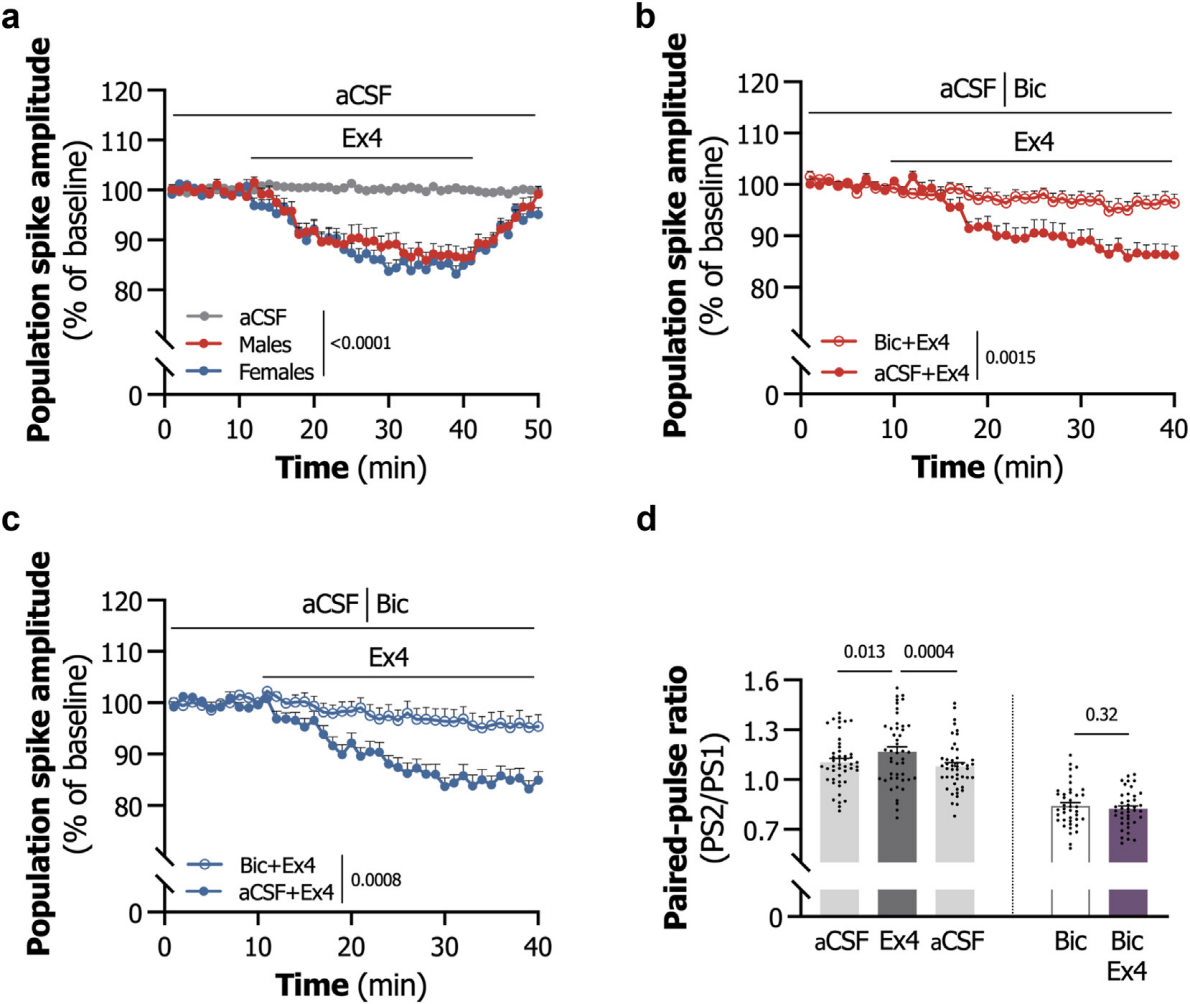
**Lateral septum GLP-1 receptor activation suppresses neurotransmission via a GABA_A_ receptor-dependent mechanism in *ex vivo* brain slices from male and female rats. a.** Application of exendin-4 (Ex4, 500 nM) significantly suppresses population spike (PS) amplitude in brain slices from both male and female rats compared to artificial cerebrospinal fluid (aCSF, n = 21–24, repeated measures two-way ANOVA). **b.** Co-application of the gamma-aminobutyric acid (GABA)_A_ receptor antagonist Bicuculline (Bic, 20 μM) with Ex4 (500 nM) attenuates the Ex4-induced suppression of population spike amplitude in male brain slices compared to aCSF (n = 19–24, repeated measures two-way ANOVA). **c.** Similarly, in female brain slices, co-application of Bic (20 μM) with Ex4 (500 nM) reduces the Ex4-induced suppression of population spike amplitude compared to aCSF (n = 21, repeated measures two-way ANOVA). **d.** Paired-pulse ratio (PPR) was increased by Ex4 (500 nM) wash-on in brain slices of alcohol-consuming rats. A significant decrease was additionally observed during the wash-out phase (n = 24, paired t-test). No statistically significant difference was noted on paired-pulse ratio when Ex4 (500 nM) was washed-on in combination with Bic (20 μM) in brain slices of alcohol-consuming rats. Data are shown as individual data points with mean ± SEM presented.

The initial stimulus–response curve indicated a statistically similar evoked response in slices from both male and female rats (F_1,109_ = 0.11, P = 0.74, repeated-measures two-way ANOVA; Supplementary Fig. S5a). Both sexes exhibited a significant reduction in population spike amplitude upon Ex4 (500 nM) application compared to aCSF (F_2,65_ = 25.51, P < 0.0001, one-way ANOVA; Fig. 6a), which was fully reversed upon drug wash-out.

Considering that changes in GABAergic transmission have been suggested as a key mechanism underlying GLP-1R effects in LS,^30^^,^^64^ slices were pretreated with the GABA_A_R antagonist bicuculline (20 μM) prior to Ex4 wash-on. Bicuculline significantly increased population spike amplitude, with a concomitant decrease in paired-pulse ratio, in slices from both male and female rats, with no statistically observed sex differences (bicuculline wash-on: F_1,41_ = 0.03, P = 0.87, repeated-measures two-way ANOVA; Supplementary Fig. S5b, male paired-pulse ratio: t_20_ = 4.43, P = 0.0003, paired t-test; Supplementary Fig. S5c; female paired-pulse ratio: t_20_ = 5.20, P < 0.0001, paired t-test; Supplementary Fig. S5d).

When bicuculline (20 μM) was combined with Ex4 (500 nM), the effect of Ex4 on population spike amplitude was attenuated. This reduction was significant in both male (F_1,43_ = 11.47, P = 0.0015, repeated-measures two-way ANOVA; Fig. 6b) and female slices (F_1,40_ = 13.11, P = 0.0008, repeated-measures two-way ANOVA; Fig. 6c) compared to Ex4 alone.

Ex4 wash-on was associated with an increase in paired-pulse ratio in aCSF-treated slices (t_44_ = 2.60, P = 0.013, paired t-test), but not in slices pre-treated with bicuculline (t_44_ = 3.83, P = 0.32, paired t-test). In addition, the increase in paired-pulse ratio returned to baseline levels upon drug wash-out (t_41_ = 1.01, P = 0.0004, paired t-test; Fig. 6d), demonstrating that the acute effect by GLP-1R activation can be fully reversed.

## Discussion

This study aimed to elucidate the role of LS GLP-1R in mediating alcohol-induced reward and alcohol consumption. Here, we report that LS GLP-1R is involved in mediating the effects of systemically administrated GLP-1R agonist Ex4, specifically in its ability to suppress the stimulatory properties of alcohol. Intra-LS infusion of Ex4 reduced the rewarding dopaminergic effects of alcohol and decreased alcohol intake in a dose-dependent manner, without affecting food or water consumption. Additionally, inhibition of LS GLP-1R specifically increased alcohol consumption, but not food or water intake. Notably, we observed potential sex differences, with elevated LS GLP-1R expression correlating with higher alcohol intake in male rats, but not in females when compared to low alcohol-consuming animals. Furthermore, LS GLP-1R signalling suppressed neuronal activity *ex vivo* in a presynaptic GABA_A_R-dependent manner. Collectively, these results suggest that LS GLP-1R inhibit alcohol consumption by modulating dopaminergic reward processes potentially through a GABA_A_-dependent mechanism.

Our initial findings indicate a role for LS GLP-1R in mediating the systemic effects of Ex4 on alcohol-related behaviours. The potential importance of this role is further supported by the fact that systemically administered GLP-1R agonists, such as Ex4 and semaglutide, likely reach the LS, possibly via the adjacent subfornical organ.^30^^,^^65^^,^^66^ However, it should be noted that other brain regions, such as the nucleus of the solitary tract (NTS), are also involved in mediating the central effects of systemic GLP-1R agonists,^69^ not just the LS. These findings did however warrant further exploration of GLP-1R signalling within the LS, particularly in the context of alcohol-related behaviours.

Our data demonstrate that intra-LS administration of Ex4 effectively attenuated the rewarding properties of alcohol, as evidenced by reduced locomotor stimulation, place preference, and accumbal dopamine release in male rodents. The LS has in recent years resurfaced as an important regulatory hub for reward and motivated behaviours,^48^, ^49^, ^50^, ^51^, ^52^ as well as being linked to the dopamine-elevating effect of alcohol in the NAc.^58^ This finding supports the hypothesis that GLP-1R signalling within the LS suppresses alcohol-induced responses by inhibiting drug-induced activation of the dopaminergic reward system. Further supporting this are the earlier findings that GLP-1R modulates dopaminergic alcohol-related responses in brain areas connected to the LS, such as the NAc, VTA, LDTg, and NTS.^26^^,^^30^^,^^31^^,^^69^ The effect on the reward system is also evident in studies on LS GLP-1R and reward-related behaviours for palatable food and cocaine.^55^^,^^56^^,^^64^^,^^86^ Additionally, Ex4 has been shown to reduce septal cue reactivity to alcohol in patients with AUD.^46^ Since the rewarding effects and experience of alcohol are associated with the risk of developing AUD^7^, ^8^, ^9^, ^10^, ^11^ we propose that LS GLP-1R signalling modulates the dopaminergic reward system to inhibit alcohol-induced reward, thereby reducing alcohol intake.

Our alcohol drinking studies further support the theory that LS GLP-1R modulates alcohol consumption tentatively through reward-related processes. Intra-LS infusion of Ex4 reduced alcohol intake in both male and female rats at all time points with similar effect size to the those in NAc, VTA, LDTg and NTS.^31^^,^^69^ These findings align with previous studies showing decreased alcohol self-administration in male mice following LS Ex4 infusion^30^ and reduced alcohol consumption with both systemic^32^, ^33^, ^34^, ^35^, ^36^, ^37^, ^38^ and locally brain targeting^30^^,^^31^^,^^69^ administered GLP-1R agonists. The clinical importance of these results is underscored by the magnitude of the observed effects. Male rats treated with 0.05 μg Ex4 consumed, on average, 28.7% less alcohol over 24 h compared to the vehicle (95% CI: −25.6 to 6.87). For females, the higher dose decreased 24-h alcohol intake by an average of 35.3% compared to the vehicle (95% CI: −41.6 to −18.5). These reductions suggest a moderate to large effect on alcohol intake, which may be clinically relevant. Importantly, these findings are in line with a recent clinical study on semaglutide and alcohol intake,^47^ highlighting the potential translational value of our preclinical research. Notably, intra-LS Ex4 did not statistically affect food or water intake during the first 4 h, indicating its selective influence on reward-driven alcohol consumption. It should however be noted that the higher dose decreased body weight and showed a trend for reduced food intake at 24 h in both sexes, indicating that additional processes, such as consummatory behaviours, might be involved after the first couple of hours. A dose–response relationship was observed in both sexes, though the timing of the most pronounced Ex4 effect differed. In males, the effect was rapid but diminished over time, whereas in females, the effect increased over time. Supporting this, sex-divergent effects of GLP-1R agonists on alcohol intake have been observed previously.^34^^,^^36^^,^^87^ While the mechanisms behind these sex differences remain unclear, several interacting factors likely contribute. Gonadal hormones, particularly oestrogen, have been shown to interact with GLP-1R function to influence food intake and reward behaviour.^70^^,^^88^, ^89^, ^90^, ^91^ Oestrous cycle phases may also affect GLP-1R signalling, and sex differences exist in responses to both systemic and local GLP-1R agonists on alcohol- and food reward-related behaviours.^34^^,^^36^^,^^70^^,^^87^, ^88^, ^89^, ^90^, ^91^ Additionally, GLP-1R expression might vary between sexes in a brain region-specific manner.^89^^,^^91^ Indeed, long-term alcohol consumption has been shown to potentially alter the peripheral GLP-1 system and central GLP-1R expression.^31^^,^^82^, ^83^, ^84^, ^85^ Which prompted us to investigate whether LS GLP-1R levels change in a similar manner. We found that elevated LS GLP-1R levels were present in high alcohol-consuming male rats but not in females, compared to low-alcohol consuming animals, suggesting sex-dependent effects. Interestingly, low alcohol-consuming animals of both sexes displayed similar GLP-1R gene expression levels, indicating that the amount of alcohol consumed could be a contributing factor. The elevated GLP-1R levels in high alcohol consuming males may be due to lower blood GLP-1 levels caused by the alcohol consumption,^82^^,^^85^ which trigger a compensatory mechanism in the receptor function. Indeed, alcohol intake have been shown to alter signalling and receptor function of several neurotransmitters including dopamine, glutamate, and GABA.^92^, ^93^, ^94^, ^95^, ^96^, ^97^ The underlying causes of these effects and differences should be explored further in future research. Elevated GLP-1R expression may indicate altered LS GLP-1R function, potentially affecting dopaminergic signalling and leading to different behavioural responses to alcohols’ rewarding effects.^7^^,^^9^^,^^11^^,^^14^^,^^16^^,^^31^

To further explore how altered LS GLP-1R function might affect alcohol intake, we conducted an additional alcohol-drinking study in which we blocked LS GLP-1R to simulate a loss-of-function. This increased alcohol intake while food or water consumption were not statistically different from the vehicle, suggesting that GLP-1R inhibition indeed enhances reward-driven alcohol consumption, further supporting our hypothesis that GLP-1R in the LS modulates reward processes related to alcohol intake. Earlier studies further support this, showing that GLP-1R antagonism increases alcohol intake and reward-related behaviours,^55^^,^^56^^,^^70^^,^^98^ and that LS GLP-1R knockout mice display heightened cocaine-seeking behaviours.^64^ Additionally, chemogenetic manipulation of the LS has been shown to increase alcohol consumption.^60^ These findings emphasise the inhibitory neuromodulatory role of LS GLP-1R in alcohol consumption, suggesting that its dysfunction, at least in males, may promote reward-related disorders.

A tentative underlying mechanisms of action across sexes was provided by our neurophysiological data as Ex4 perfusion suppressed neuronal activity similarly in both sexes, with a concomitant increase in PPR, indicating that Ex4 reduces the probability of transmitter release, via a presynaptic mechanism. This is consistent with the actions of Ex4 in other brain regions.^24^^,^^25^^,^^99^^,^^100^ Synaptic depression induced by Ex4 was attenuated in slices pre-treated with the GABA_A_R antagonist bicuculline, implying that LS GLP-1R signalling relies on GABAergic neurotransmission. This aligns with data from other brain regions, where some of the effects of GLP-1R agonists are mediated by GABA transmission,^29^^,^^37^^,^^64^^,^^72^^,^^101^, ^102^, ^103^, ^104^ specifically GABA_A_R.^72^^,^^101^^,^^103^, ^104^, ^105^ Given that the LS is primarily composed of GABAergic neurons^48^, ^49^, ^50^ which highly express GLP-1R,^18^^,^^19^^,^^21^ and that inhibition of septal neurons prevents alcohol-induced dopamine release in the NAc,^58^ GLP-1R-induced depression of excitability may be a potential mechanism underlying the effects of GLP-1R agonists on reward-related behaviours in the LS.^30^^,^^64^

Overall, our findings support and extend the inhibitory role of LS GLP-1R signalling in alcohol- and dopaminergic reward-related behaviours, potentially via a GABAergic mechanism. This aligns with the hypothesis proposed by Allingberg et al. (2023), which suggests that GLP-1R activation in the LS may modulate VTA dopaminergic neurons through direct inhibition or by influencing GABAergic neurons in the VTA or NAc. Our results provide additional evidence for this circuit, where GLP-1R activation in the LS could increase tonic inhibition of VTA dopaminergic neurons, consequently reducing alcohol-induced dopamine release in the NAc. This study offers further support for the proposed circuit involving the dense GABAergic connections from LS to NAc and VTA.^48^^,^^49^^,^^54^^,^^58^^,^^62^^,^^63^^,^^106^ To fully elucidate the exact pathways and mechanisms involved, future research should employ higher temporal resolution techniques such as optogenetics and biosensors, along with tracing studies. These approaches will be crucial in confirming and refining our understanding of the LS-VTA-NAc circuit in GLP-1R-mediated modulation of alcohol-related behaviours.

To ensure that the observed effects were specific to reward processes and not due to nonspecific alterations in general behaviour, we used doses of Ex4 consistent with previous studies.^30^^,^^55^^,^^56^ These studies have shown that such doses do not affect gross behaviour, anxiety-like behaviours, or malaise, a common side effect of GLP-1R agonists in both rodents and humans.^14^^,^^16^ Supportively, we found that LS GLP-1R antagonism did not influence locomotor behaviour by itself in the initial study, and we observed no effect of Ex4 on alcohol-induced locomotion in ICV- or CPu-injected animals, suggesting that potential diffusion of Ex4 from the LS to adjacent areas do not contribute to the effect, which have also been shown before with fluorescence.^30^^,^^55^ Additionally, intra-LS Ex4 did not influence place conditioning by itself in the CPP paradigm. Importantly, our microdialysis experiment show that local administration of Ex4 in LS did not affect baseline dopamine levels in the NAc. The counterbalanced design in the alcohol drinking studies where rats served as their own controls, made it possible to compare alcohol intake between rats receiving Ex4 on test day one and vehicle on test day two against baseline levels. Alcohol consumption returned to baseline in all four drinking experiments, indicating that intra-LS Ex4 did not induce taste aversion to alcohol. Collectively, these findings suggest that the effect of Ex4 on alcohol-related responses in the LS is specific to reward processes.

However, our study has some limitations. First, the absence of alcohol-naïve water-drinking controls in both electrophysiological and gene expression studies is a potential confounding factor, as differences between groups may exist independently of alcohol, which should be accounted for in future studies. Second, in our electrophysiology experiments, we tested only one Ex4 concentration. While this concentration aligns with previous studies,^24^^,^^25^^,^^28^^,^^71^^,^^72^ testing additional concentrations is warranted to confirm that it is appropriate for use in the LS. Furthermore, the observed sex differences merit more exploration to determine involved mechanisms. For instance, testing whether Ex9 also increases alcohol intake in females, which were not investigated here. Additionally, potential effects on nausea, stress and anxiety, which are known effects of GLP-1R agonists,^14^^,^^16^ were not assessed, which could have influenced the statistical outcomes. A notable limitation of within-group comparisons, used in the drinking experiments in this study, is that observed effects might be influenced by both regression to the mean and time-related factors, rather than solely Ex4 infusion effects. While our counterbalanced design helps mitigate some of these concerns, future studies using between-group comparisons could further validate these findings. While this study also did not address the motivational aspects of reward linked to LS,^49^^,^^51^ future research should explore this area more thoroughly. Finally, pharmacological manipulation of the GLP-1–GABA_A_ link could provide new insights into the effects of LS GLP-1R signalling on alcohol and other substances of abuse, such as nicotine and opioids. Investigating these effects in the context of repeated drug exposure could reveal broader implications, particularly concerning behavioural sensitisation, cue reactivity, reinstatement, and craving, which have been linked to LS.^48^^,^^49^^,^^51^^,^^54^^,^^64^

In conclusion, GLP-1R agonists are emerging as potential pharmacotherapeutic candidates for AUD. Although the underlying neurobiological mechanisms involved remain to be elucidated, our study provides new insights into how GLP-1R agonists act in the brain. We propose that LS GLP-1R signalling has a modulatory role in alcohol consumption by mediating dopaminergic reward processes through a presynaptic GABA_A_R-dependent mechanism. The observed sex-specific differences in LS GLP-1R effect also emphasise the need for further research into treatment response variations between sexes, which could potentially enhance therapeutic outcomes. Further research is needed to clarify these mechanisms and their potential applications in AUD and tentatively, other substance use disorders.

## Contributors

**Christian E Edvardsson:** Conceptualisation, Methodology, Investigation, Formal analysis, Data curation, Writing—original draft, Writing—review & editing, Visualisation, Project administration, Validation, Data verification. **Davide Cadeddu:** Investigation, Formal analysis, Data curation, Writing—review & editing, Validation, Data verification. **Mia Ericson:** Writing—review & editing, Resources, Validation. **Louise Adermark:** Conceptualisation, Methodology, Formal analysis, Data curation, Writing—review & editing, Resources, Validation, Data verification. **Elisabet Jerlhag:** Conceptualisation, Formal analysis, Writing—review & editing, Resources, Funding acquisition, Validation, Data verification.

All authors have read and approved the final version of the manuscript.

## Data sharing statement

Data collected for the study will be made available and shared with others though contact at the following address: elisabet.jerlhag@pharm.gu.se. The data will be shared with researchers who want to do additional analysis of the data and therefore the data will be shared after approval of a proposal, and with a signed data access agreement.

## Declaration of interests

EJ received paid travel expenses for keynote lecture at the conference ISBRA 2024. CEE, LA, ME and DC have no conflicts of interests to declare.
