# Supplementary material for: An inhibitory GLP-1 circuit in the lateral septum modulates reward processing and alcohol intake in rodents
Source: eBioMedicine. 2025 Apr 17;115:105684. doi: 10.1016/j.ebiom.2025.105684 (PMC12044336; doi:10.1016/j.ebiom.2025.105684)
Supplement: Supplementary Figs. S1–S6 [file mmc1.docx]

**Figure S1 (Supplementary Figure 1)**

**
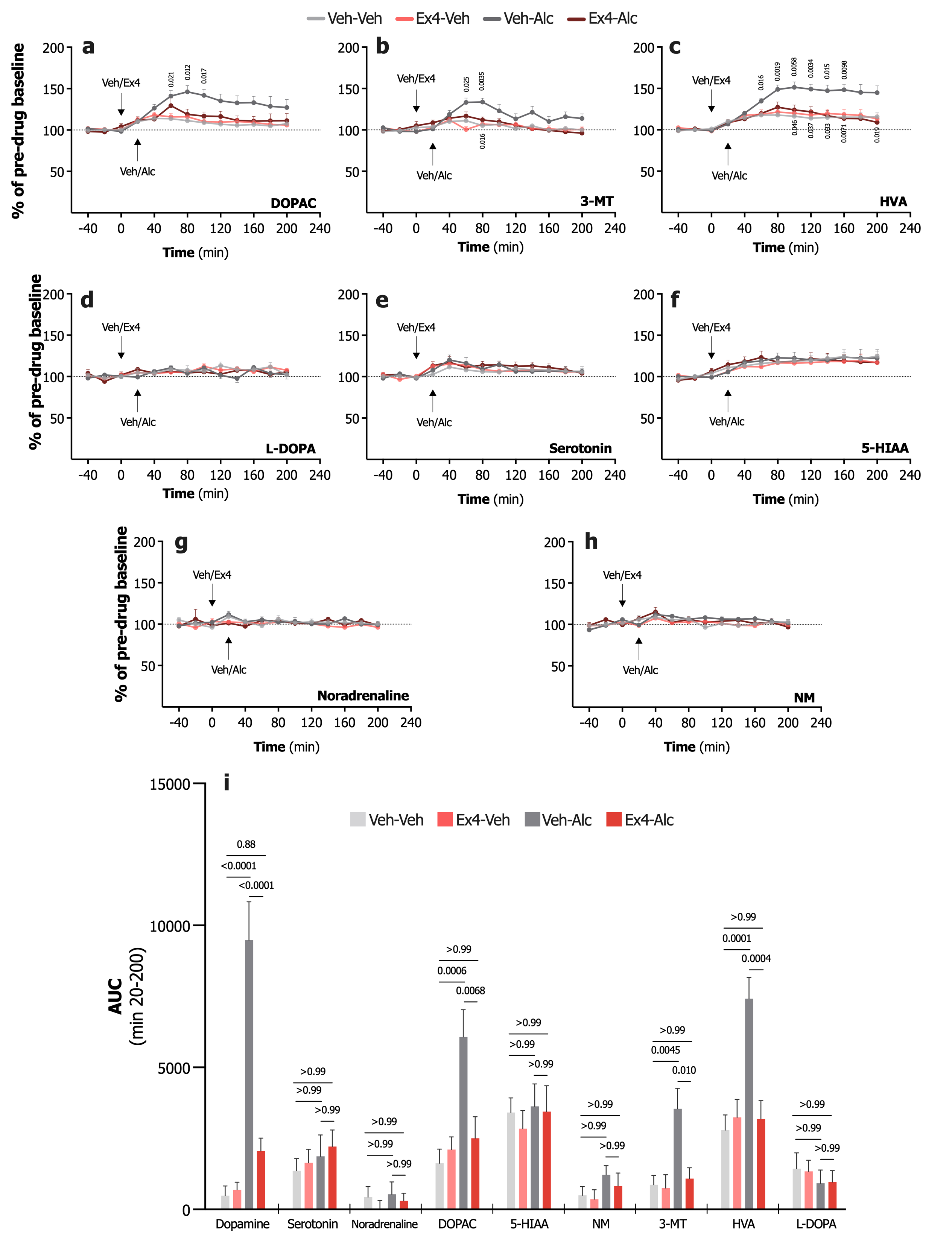
**

**Figure S1. Accumbal monoamine levels in male rats following systemic alcohol administration and infusion of exendin-4 into the lateral septum.**

**a-c.** Compared to vehicle (Veh), alcohol (Alc, 1.75 g/kg, IP) evoked a release of 3,4-dihydroxyphenylacetic acid (DOPAC), 3-methoxy-4-hydroxyphenethylamine (3-MT), and homovanillic acid (HVA) in the nucleus accumbens shell (NAcS), effects significantly attenuated by infusion of exendin-4 (Ex4) (0.05 µg) into lateral septum of male rats (n=8, repeated measures two-way ANOVA). **d-h.** No statistical differences were observed in the levels of noradrenaline, normetanephrine (NM), serotonin, 5-hydroxyindoleacetic acid (5-HIAA), or L-β-3,4-dihydroxyphenylalanine (L-DOPA) in the NAcS during microdialysis in male rats (n=8, repeated measures two-way ANOVA). **i.** Similar effects were also reflected in the area under the curve (AUC) analysis (n=8, one-way ANOVA). Data are presented as mean±SEM.

**Figure S2 (Supplementary Figure 2)**

**
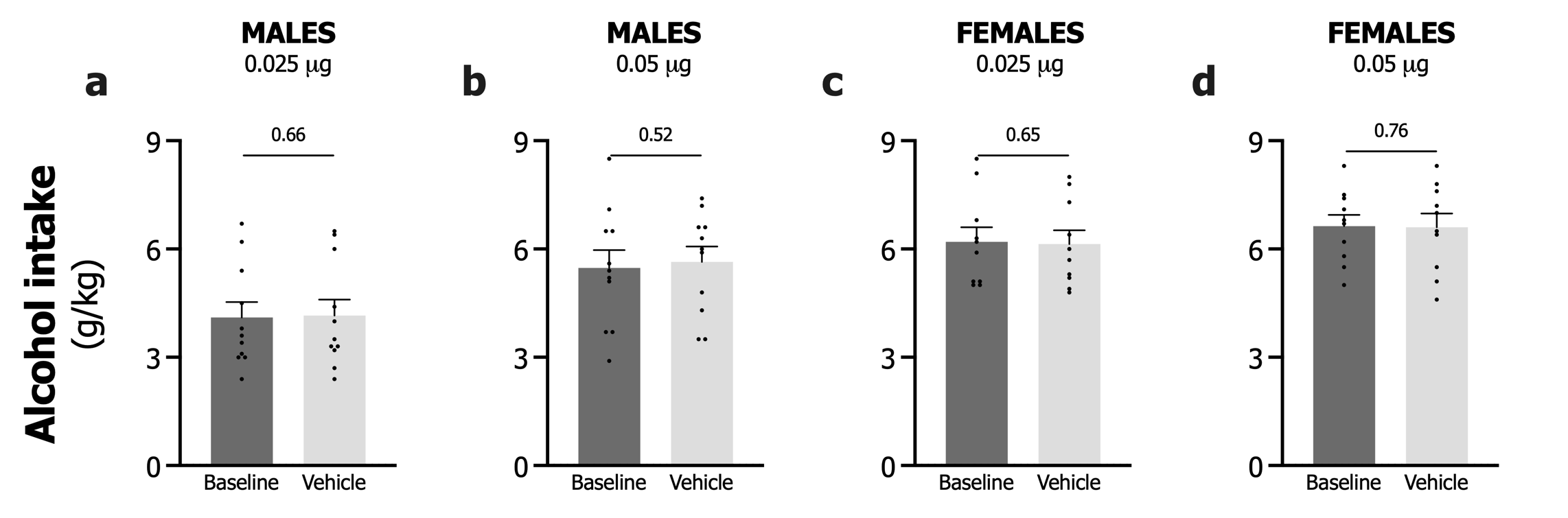
**

**Figure S2. Comparison between baseline and vehicle into lateral septum.**

**a-d.** The alcohol intake levels for the vehicle animals were at similar levels to baseline intake across all test days for both sexes in all four drinking studies. **a.** Males 0.025 μg Ex4 (t_10_=0.45, P=0.66), **b.** males 0.05 μg Ex4 (t_10_=0.67, P=0.52), **c.** females 0.025 μg Ex4 (t_9_=0.47, P=0.65) and **d.** females 0.05 μg Ex4 (t_9_=0.32, P=0.76, n=10-11, paired t-test). Data are shown as individual data points with mean ± SEM presented.

**Figure S3 (Supplementary Figure 3)**

**
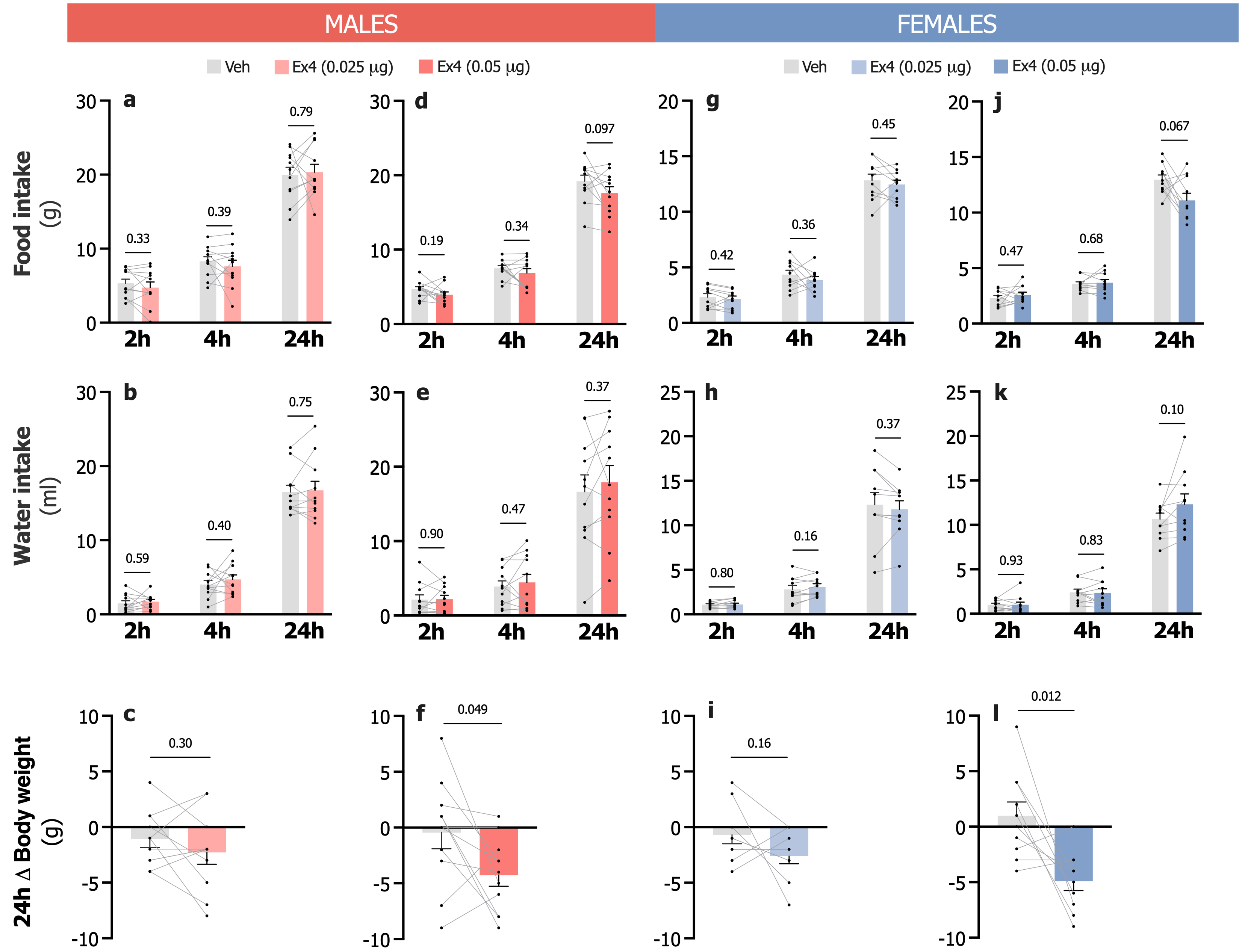
**

**Figure S3. Effects of activation of GLP-1 receptors in lateral septum on food intake, water intake, and body weight in male and female rats.**

**a-c.** There is no statistical difference after intra-lateral septum (LS) infusion of 0.025 µg exendin-4 (Ex4) on food intake (2h t_10_=1.02, P=0.33, 4h t_10_=0.90, P=0.39, 24h t_10_=0.28, P=0.79), water intake (2h t_10_=0.56, P=0.59, 4h t_10_=0.87, P=0.41, 24h t_10_=0.75, P=0.33), or body weight (t_10_=1.09, P=0.30) at 2, 4, and 24 hours compared to vehicle (Veh) in male rats (n=11, paired t-test). **d-f.** There is no statistical difference after intra-LS infusion of 0.05 µg Ex4 on food (2h t_10_=1.42, P=0.19, 4h t_10_=1.01, P=0.34, 24h t_10_=1.83, P=0.097) or water (2h t_10_=0.13, P=0.90, 4h t_10_=0.75, P=0.47, 24h t_10_=0.93, P=0.37) intake at 2, 4, and 24 hours compared to vehicle; however, a decrease in body weight (P=0.049) is observed in male rats (n=11, paired t-test). **g-i.** Similarly, intra-LS infusion of 0.025 µg Ex4 is not statistically different for food intake (2h t_9_=0.84, P=0.42, 4h t_9_=0.96, P=0.36, 24h t_9_=0.78, P=0.45), water intake (2h t_9_=0.26, P=0.80, 4h t_9_=1.53, P=0.16, 24h t_9_=0.94, P=0.37), or body weight (t_9_=1.55, P=0.16) at 2, 4, and 24 hours compared to vehicle in female rats (n=10, paired t-test). **j-l.** There is no statistical difference in intra-LS infusion of 0.05 µg Ex4 on food (2h t_9_=0.75, P=0.47, 4h t_9_=0.43, P=0.68, 24h P=0.067) or water (2h t_9_=0.09, P=0.93, 4h t_9_=0.22, P=0.83, 24h P=0.10) intake at 2, 4, and 24 hours compared to vehicle; however, a decrease in body weight (t_9_=3.19, P=0.012) is observed in female rats (n=10, paired t-test). Data are shown as individual data points with mean ± SEM presented.

**Figure S4 (Supplementary Figure 4)**


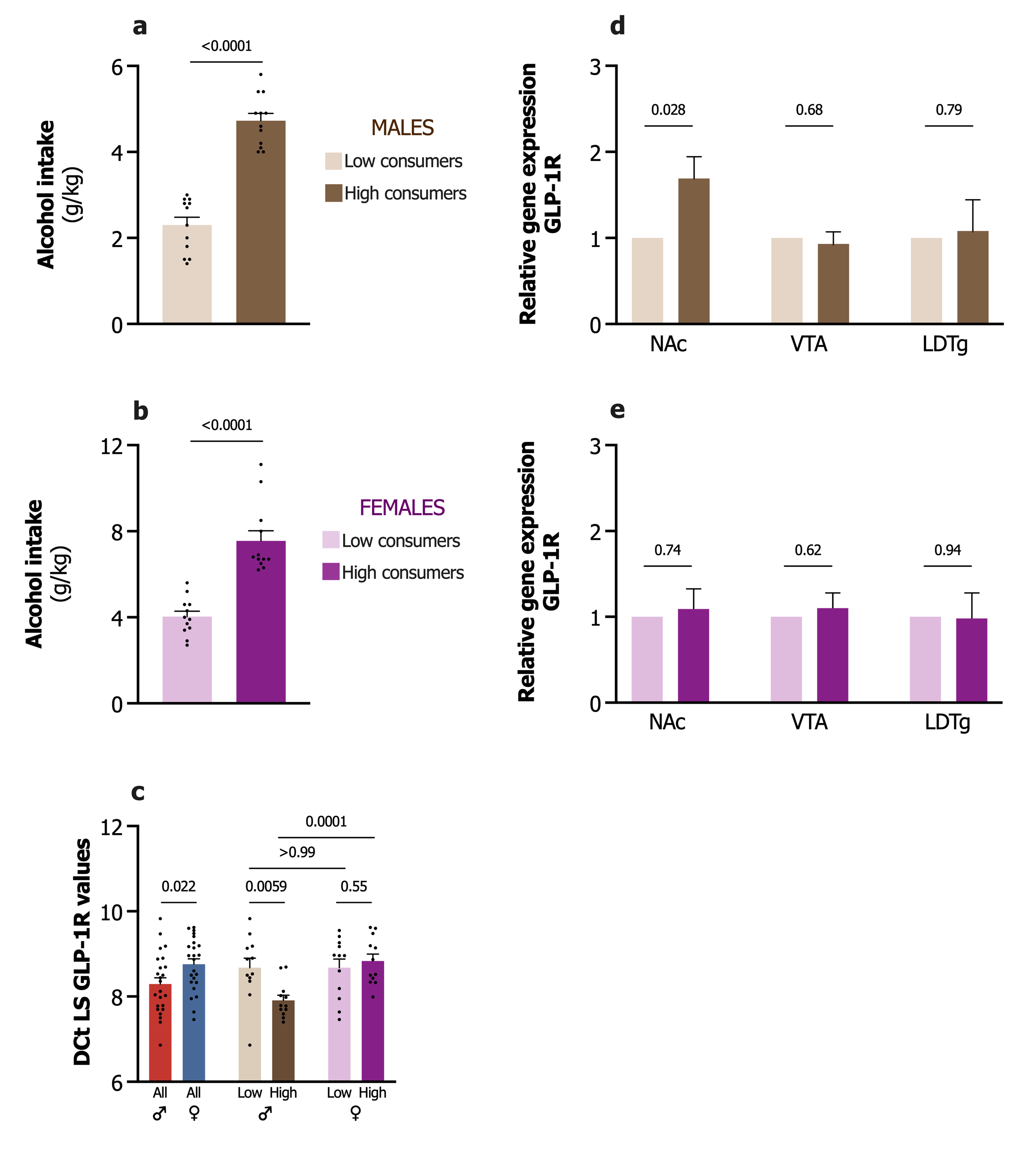


**Figure S4. GLP-1 receptor expression in male and female rats following long-term alcohol consumption in reward-associated brain areas.**

**a.** Alcohol intake was significantly different between low and high alcohol-consuming male rats used for GLP-1 receptor (GLP-1R) expression analysis (n=12; t_22_=9.70, P<0.0001, unpaired t-test). **b.** Alcohol intake was significantly different between high and low alcohol-consuming female rats used for GLP-1R expression analysis (n=12; t_22_=6.76, P<0.0001, unpaired t-test). **c.** The delta threshold cycle (DCt) GLP-1R expression values in lateral septum (LS) were significant between sexes (n=24, t_46_=2.37, P=0.022, unpaired t-test). An effect that appears to be driven by the high alcohol-consuming males (n=12, t_22_=3.05, P=0.0059, unpaired t-test) compared to the low consuming. No statistical difference was observed between the low alcohol-consuming male and female animals. **d.** GLP-1R expression significantly increases in the nucleus accumbens (NAc) (n=12; t_22_=2.36, P=0.028, unpaired t-test of DCt values) of high alcohol-consuming male rats compared to low alcohol-consuming rats, shown as relative gene expression normalized to the low consumers. No statistical differences were observed in the ventral tegmental area (VTA; t_22_=0.42, P=0.68) or the laterodorsal tegmental area (LDTg; t_22_=0.27, P=0.79). **e.** No statistical differences were observed in GLP-1R expression in the NAc (t_22_=0.33, P=0.74), VTA (t_22_=0.50, P=0.62), or LDTg (t_22_=0.07, P=0.94) between high and low alcohol-consuming female rats, presented as relative gene expression normalized to the low consumers (n=12; unpaired t-test of DCt values). Data are presented as mean±SEM.

**Figure S5 (Supplementary Figure 5)**

**
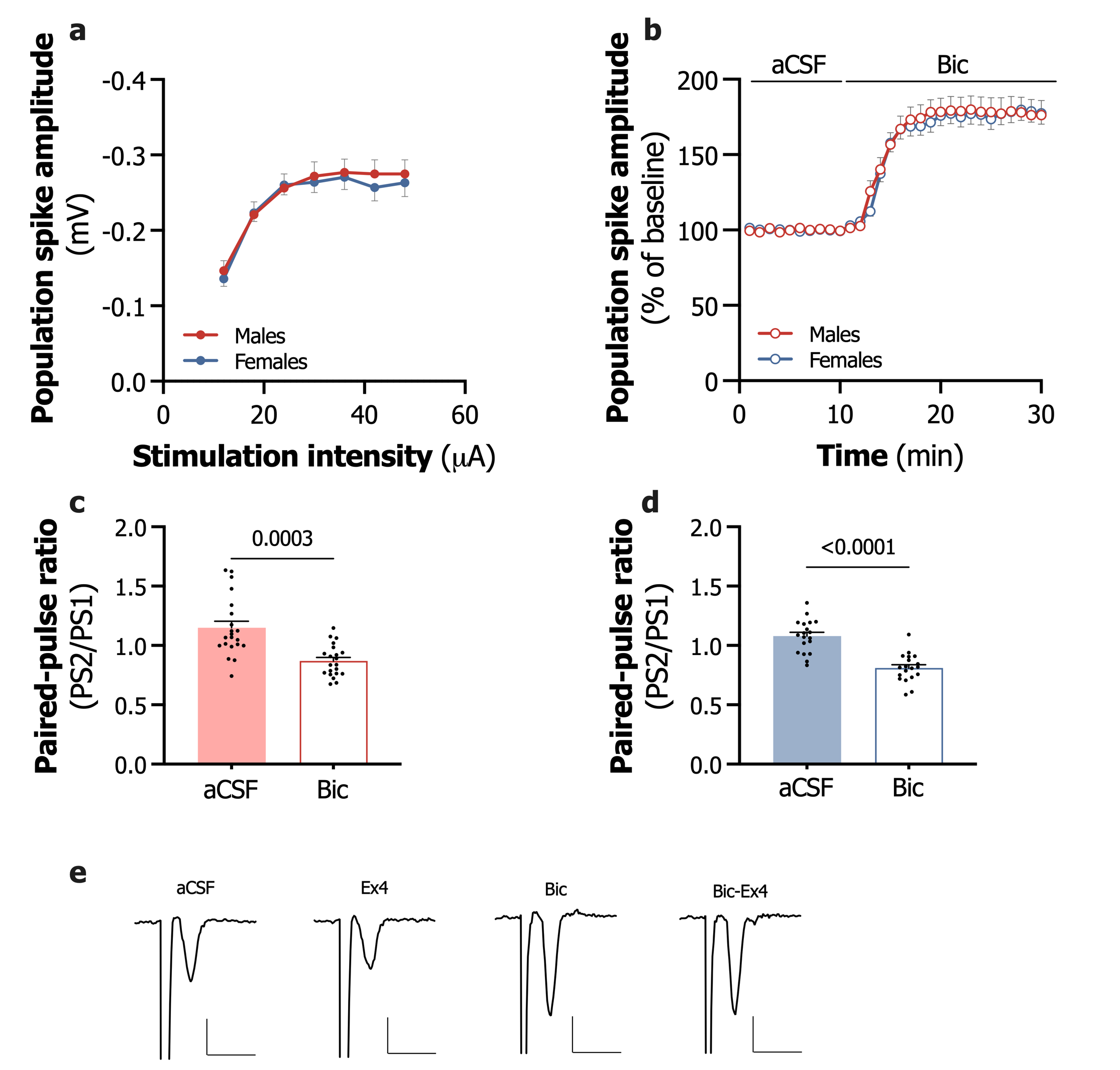
**

**Figure S5. Additional effects on lateral septum neurotransmission in male and female rats.**

**a.** No statistical differences were observed between male and female brain slices of lateral septum (LS) in the stimulus-response curve (n=55, P=0.73, repeated measures two-way ANOVA). **b.** There were no statistical sex differences in the increase in population spike amplitude following the application of the gamma-aminobutyric acid (GABA)_A_ receptor antagonist Bicuculline (Bic, 20 µM) in male and female brain slices compared to artificial cerebrospinal fluid (aCSF; n=21, P=0.87, repeated measures two-way ANOVA). **c-d.** Bic decreases paired-pulse ratio in male (aCSF-Bic, P=0.0003) and female (aCSF-Bic, P<0.0001) slices (n=21; paired t-test). **e.** Example traces demonstrating evoked field potentials in the LS during aCSF, exendin-4 (Ex4), Bic and Bic-Ex4 wash-on. Calibration: 0.1 mV, 2 ms. Data are presented as mean±SEM.
